# Ear pinna growth and differentiation is conserved in murids and requires BMP signaling for chondrocyte proliferation

**DOI:** 10.1242/dev.204560

**Published:** 2025-02-13

**Authors:** Robyn S. Allen, Shishir K. Biswas, Ashley W. Seifert

**Affiliations:** Department of Biology, University of Kentucky, Lexington, KY 40506, USA

**Keywords:** Ear pinna, Neural crest cells, Cell lineage, BMP5, *Short ear*, *Acomys*, Elastic cartilage

## Abstract

Despite being a major target of reconstructive surgery, development of the ear pinna remains poorly studied. Here, we provide a cellular characterization of late gestational and postnatal ear pinna development in two rodents and investigate the role of BMP5 in expansion and differentiation of auricular elastic cartilage. We find that ear pinna development is largely conserved between *Mus musculus* and the highly regenerative *Acomys dimidiatus*. The pattern of pre-cartilaginous cells is established early in development. These cells are specified into chondroblasts before ear unfolding and then undergo extensive proliferation before maturation. The elastic cartilage, connective tissue fibroblasts, dermal papilla and sheath cells, and adipocytes in the adult pinna are derived from cranial neural crest. Cellular analysis using the naturally occurring *short ear* mouse mutant shows that loss of BMP5 does not prevent specification of chondroblasts, but does impair chondroblast proliferation. Finally, chondroblast proliferation remains impaired in the adult mid-distal ear pinna of these mutants. Together, these data establish the developmental basis for differentiation of ear pinna tissues.

## Teaser

**Summary:** Comparative histology of murids, genetic lineage tracing, and characterization of the *short ear* mouse strain reveals the cellular origin and maturation sequence of ear pinna tissues.

## INTRODUCTION

Craniofacial tissues are a major target of reconstructive surgeries and bioengineering approaches to repair developmental defects and tissue injuries (Habal, 2004; Haisch, 2010; Schantz et al., 2012; Cubitt et al., 2019; Jia et al., 2022). The ear pinna (auricle) is a part of the cranio-facial complex and plays an important role in directing sound into the middle and inner ear for hearing. Injury or developmental perturbation to the ear pinna can impair hearing and create aesthetic outcomes that lead to psychological distress in patients. The ear pinna also represents an excellent model for evolutionary, developmental, and regeneration studies due to a highly conserved anatomical structure (Chiu et al., 2017). While development of the inner and middle ear has been well-studied in mammals (Anthwal and Thompson, 2016; Whitfield, 2015), external ear development is poorly understood.

Available developmental data of the ear pinna has focused on early stages of development and comes from limited observations of human and mouse embryos (Cox et al., 2014; Green and Green, 1942; Hashimoto et al., 2021; Honkura et al., 2020; Hunter and Yotsuyanagi, 2005; Mallo and Gridley, 1996; Minoux et al., 2013; Theiler and Sweet, 1986). In humans, the Hillocks of His contribute to the pinna components, though the exact sources of these cells within the pharyngeal arches is debated (Green and Green, 1942; Theiler and Sweet, 1986; Cox et al., 2014; Palmer et al., 2016). In mouse, genetic fate mapping (Minoux et al., 2013) as well as loss- and gain-of-function studies (Rijli et al., 1993; Mallo and Gridley, 1996; Minoux et al., 2013) demonstrated that the ear pinna, with the possible exception of the tragus, is derived from the Hoxa2^+^ cranial neural crest cells of the second pharyngeal arch and that, in these cells, Hoxa2 is required for pinna morphogenesis. While these studies provide important insight into early pinna development, further characterization of tissue differentiation and maturation is necessary to understand morphogenesis of the adult structure.

The bone morphogenetic protein (BMP) family ligands, BMP4 and BMP5, are under *Hoxa2* regulation and play an important role in the development of auricular elastic cartilage (King et al., 1994; Minoux et al., 2013; Yang et al., 2024). Perturbations to the BMP signaling pathway underlie some ear pinna developmental defects in humans and mice, though many questions remain regarding their specific roles (King et al., 1994; Liu et al., 2022; Luquetti et al., 2012; Yang et al., 2024; Zhang et al., 2020). Some studies have identified a role for BMPs in maintaining cartilage identity (Yang et al., 2024), while microtia defects from *Bmp5* mutations suggest a role in cell specification or proliferation (King et al., 1994; Liu et al., 2022). Elucidating the process of elastic cartilage differentiation will be crucial for tissue engineering approaches (Habal, 2004; Haisch, 2010; Jia et al., 2022).

The ear pinna also represents a powerful model for studying complex tissue regeneration (Seifert et al., 2012; Sosnowski et al., 2022). Wounding studies in cranial neural-crest-derived dermis revealed a unique gene expression and extracellular matrix composition compared to trunk dermis (Usansky et al., 2021). Indeed, these features may contribute to scar resistance in the craniofacial complex. Further, the conserved anatomy, and the accessibility of the ear pinna, has made it possible to screen different mammalian species for regenerative ability using a simple ear punch assay (Ekdale, 2016; Gawriluk et al., 2016; Williams-Boyce and Daniel, 1986). These studies led to the discovery that spiny mice (*Acomys spp.*) are capable of regenerating full-thickness skin and the complex musculoskeletal architecture of the ear pinna (Gawriluk et al., 2016; Matias Santos et al., 2016; Seifert et al., 2012). Understanding ear pinna development can provide deeper insight into specific cellular mechanisms that may be curtailed in non-regenerating laboratory mice and rats.

To provide a foundation to better understand normal ear pinna development, embryonic malformations, and pinna regeneration, we sought to characterize and compare ear pinna differentiation and growth in *Acomys dimidiatus* and *Mus musculus*. Using the framework generated by this comparison, we then investigated elastic cartilage development in the *short ear* mouse mutant (*Bmp5^se/se^* mutation; p.R208*), which develops truncated, malformed ears. We show that tissue maturation in the ear pinna follows the same pattern in *Acomys and Mus*. The elastic cartilage sheet differentiates as vessels and nerves grow and extend into foramina, resulting in a ‘Swiss cheese’ pattern through the cartilaginous sheet. Using genetic tools available for *Mus*, we show that neural crest cells contribute to most adult pinna tissues, including the cartilage, apart from: (1) the epidermis and epidermally derived organs, (2) skeletal muscle and (3) a sparse population of cells throughout the connective tissue. Finally, we used the naturally occurring *short ear* mutant mouse to show that BMP signaling is required for the proliferation of chondroprogenitors in the mid-distal ear and this requirement is retained into adulthood.

## RESULTS

### Ear pinna tissue architecture is conserved across murid subfamilies

**Fig. 1.**
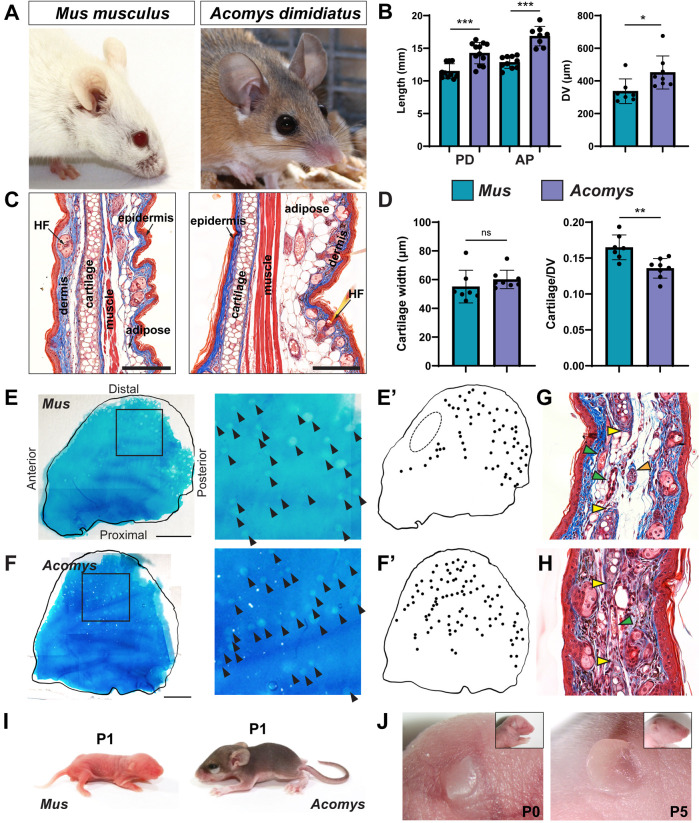
**Murid ear pinna structure is conserved.** (A) *Mus musculus* and *Acomys dimidiatus* ear pinnae. (B) Average length of the proximodistal (PD), anteroposterior (AP), and dorsoventral (DV) axes of the ear pinna from adult *Mus* (teal) and *Acomys* (purple). (C) Masson's trichrome staining of *Mus* (left) and *Acomys* (right) ear pinna sectioned along the AP axis, oriented proximal side down and dorsal side right. (D) Average cartilage width and proportion of cartilage to ear DV width in *Mus* and *Acomys* ear pinnae. (E,F) Alcian Blue staining of adult *Mus* (E; *n*=3) and *Acomys* (F; *n*=3) ear pinna cartilage. Arrowheads indicate foramina. Images on right show magnification of boxed areas on left. (E′,F′) Schematic representation of foramina in the adult *Mus* (E′) and *Acomys* (F′) auricular cartilage. Dashed oval indicates anterior area of uninterrupted cartilage (E′). (G,H) Trichrome sections of adult *Mus* (G) and *Acomys* (H) ears showing foramen (yellow arrowheads) conveying vasculature (green arrowheads) and nerves (orange arrowhead). (I) Newborn *Mus* (left) and *Acomys* (right). (J) *Mus* ear anatomy at P0 (left) and P5 (right). *P<0.05, **P<0.01, ***P<0.001 (unpaired two-tailed Student's *t*-test). Error bars show s.d. Scale bars: 100 µm (C); 3 mm (E,F).

Ear pinna anatomy is conserved between murids. Using sexually mature *Acomys dimidiatus* (≥6 months) and outbred laboratory *Mus musculus* (*ND4* strain; ≥3 months) as example murids, we compared characteristics of the adult ear pinna (Fig. 1A). First, we observed that *Acomys* ears were significantly longer than *Mus* in the proximodistal (PD), anteroposterior (AP), and dorsoventral (DV) axes (Fig. 1B). However, the tissue architecture pattern of the adult ear pinna was nearly identical between species (Fig. 1C). Both species contained a central sheet of elastic cartilage with skeletal muscle on the dorsal aspect. Adipose tissue, dermis, hair follicles, glands, and epidermis were visible on both sides of the cartilage with the dorsal side being thicker in both species. There was no significant difference in the DV width of the cartilage between the two species, but the proportion of cartilage across the DV axis was significantly greater in *Mus* (Fig. 1D). The additional DV width in *Acomys* appeared to result from adipose tissue (Fig. 1C). Using whole mount Alcian Blue staining, we were able to visualize the cartilage sheet (Fig. 1E,F; Fig. S1A,B). We observed foramina scattered throughout the elastic cartilage, creating a ‘Swiss cheese’ pattern. In *Mus*, the foramina occurred more densely in the posterior ear, with an area of solid cartilage along the anterior edge (Fig. 1E′), whereas in *Acomys* the foramina were more evenly distributed (Fig. 1F′). Histological examination showed that nerves and vasculature were present within the foramina of both species (Fig. 1G,H).

Given anatomical similarities in the ear pinnae between species, we examined postnatal *Mus* and *Acomys* ears. *Acomys* are precocial, with a gestational period twice as long as *Mus* (38-40 days versus 20-21 days) (Fig. 1I) (Brunjes, 1990; Haughton et al., 2016). Thus, a portion of ear pinna development that occurs postnatally in *Mus* occurs neonatally in *Acomys* (Fig. 1I). *Mus* are born with their pinna folded against the head, which later unfolds at postnatal day (P)5 (Fig. 1J). In contrast, *Acomys* have unfolded ears, hair follicles, and more developed ear ridges at parturition (Fig. 1I).

### Ear pinna tissue differentiation is conserved between *M. musculus* and *A. dimidiatus*

**Fig. 2.**
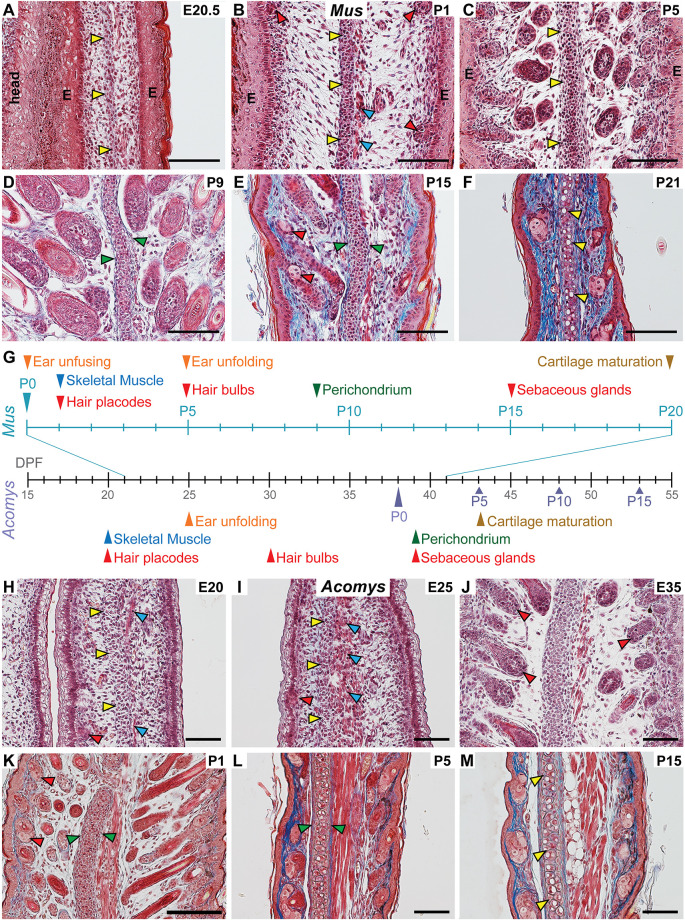
**Ear pinna differentiation is conserved between murids.** (A-F) Trichrome staining of *Mus* ear pinnae at various developmental time points. Arrowheads indicate anatomical structures that become identifiable (as differentiating tissues): cartilage (yellow), skeletal muscle (blue), perichondrium (green), hair bulbs, hair follicles, and sebaceous glands (red). (G) Stage at which differentiated tissues are identifiable in *Mus* (above) and *Acomys* (below) ear pinnae based on histological survey. Black timeline shows days post fertilization (DPF). Parturition is ∼21 dpf in *Mus* and ∼38 dpf in *Acomys*. Teal timeline shows *Mus* postnatal time points. Purple arrowheads indicate *Acomys* postnatal time points. (H-M) Trichrome staining of *Acomys* ear pinnae at various developmental time points. All sections are oriented dorsal side right and proximal side down. *Mus*; *n*=3 for all time points. *Acomys*; *n*=2 (E20-E35), *n*=3 (P1-P15). Arrowhead colors same as in A-F. Scale bars: 100 µm.

We performed a longitudinal study of ear pinna development in outbred strains of *Mus* beginning at embryonic day (E)20.5 and in *Acomys* at E20. Tissue was collected every 2-5 days until the ear had established its mature histological architecture (Fig. 2A-F,H-M; Figs S2 and S3). In *Mus*, tissue differentiation largely occurred postnatally. At E20.5 the ear pinna was folded ventrally, and we observed mesenchymal cells aggregating in the center of the ear pinna (Fig. 2A,G; Fig. S2). The pinna epidermis resembled a transitional epithelium with a stratum corneum, but the stratum spinosum and stratum basale appeared to be poorly differentiated (Fig. 2A). At P1, condensing mesenchymal cells formed a dense aggregation (hereafter referred to as the central condensation), while the dermal compartment expanded resulting in decreased cellular density (Fig. 2B). The epidermis was fully stratified, with a differentiated stratum spinosum and a stratum basale with apical-basal polarity (Fig. 2B). This transition of the epidermis coincided with the onset of hair follicle morphogenesis (Fig. 2B,G). Striated skeletal muscle was visible dorsal to the central condensation (Fig. 2B,G). Hair follicle morphogenesis continued at P5 with extension of follicles into the dermis coincident with ear unfolding (Fig. 2C,G; Fig. S2). Perichondrium and adipose became identifiable at P9 (Fig. 2D,G). Hair follicle morphogenesis continued through the second week and sebaceous glands were visible by P15 (Fig. 2E,G) (Müller-Röver et al., 2001; Paus et al., 1999). As the hair follicles completed catagen, cartilage continued maturing such that by P21 lacunae could be observed in the matrix (Fig. 2F,G). By P21 the tissue architecture in *Mus* reflected the adult condition except for complete maturation of the elastic cartilage. The DV width of the ear peaked at P9 once most tissues had differentiated (Fig. S4A). The cartilage width nominally trended up until the end of development (Fig. S4B). As the ear thinned, the proportion of cartilage within the DV width increased (Fig. S4C).

In *Acomys*, the development of the primary tissue compartments (epidermis, hair follicles, muscle, and cartilage) largely mirrored that of *Mus*, though most tissues differentiated *in utero* (Fig. 2G). As seen in *Mus*, a pre-cartilaginous mesenchymal condensation formed before tissue differentiation (Fig. 2G,H; Fig. S3). Ear unfolding occurred *in utero* between E20 and E25 when striated skeletal muscle and early hair follicle morphogenesis were identifiable (Fig. 2G-I; Fig. S3). Between E30 and E35, hair bulbs differentiated (Fig. 2G,J). At P1, the perichondrium became evident and sebaceous glands began forming (Fig. 2G,K). Maturing cartilage with lacunae was evident at P15 (Fig. 2G,L,M). Like *Mus*, ear DV width peaked around the time most tissues had achieved a mature appearance (∼P1) (Fig. S4A,B). Interestingly, the proportion of cartilage contributing to DV width was much higher at P15 compared to mature *Acomys*, likely due to post-developmental accumulation of adipose tissue (Fig. S4C). Together, our analysis provides a general scheme for ear pinna development across murids. These data also support that, while specific developmental benchmarks vary between species, the order is highly conserved (Fig. 2G).

### Elastic cartilage differentiates in parallel with other tissues

**Fig. 3.**
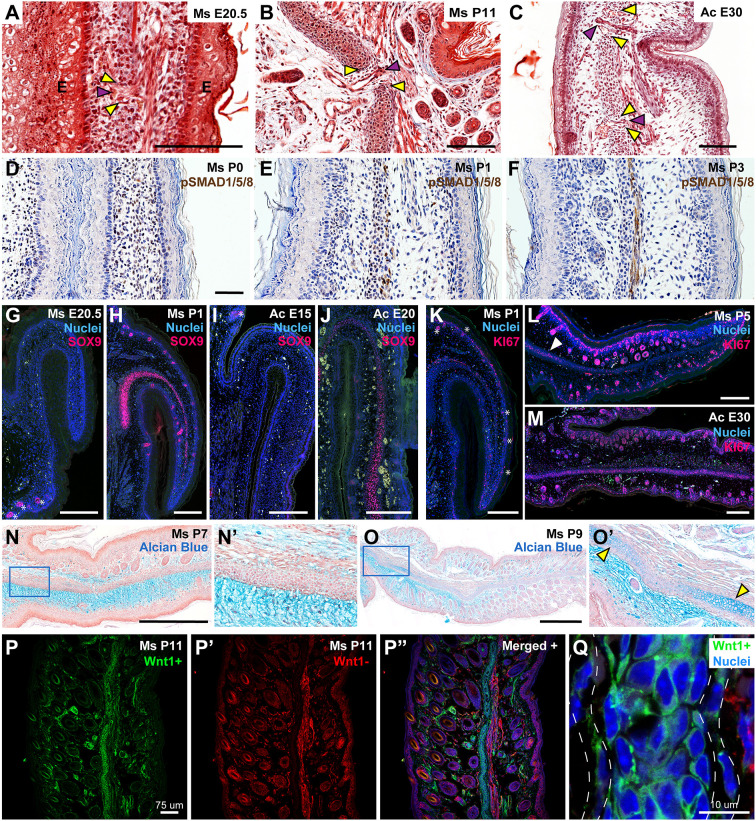
**Stages of elastic cartilage differentiation in murids.** (A-C) Trichrome staining of *Mus* (Ms) and *Acomys* (Ac) ear pinnae showing interruptions in central condensations and maturing cartilage (yellow arrowheads) containing mesenchymal tissue and maturing vasculature (purple arrowheads). (D-F) pSMAD1/5/8 immunostaining in developing *Mus* ears. (G-J) SOX9 immunostaining in *Mus* (G,H) and *Acomys* (I,J) ears at various time points. Green channel is autofluorescence. Asterisks (G,I) indicate follicles. (K-M) KI67 immunostaining in *Mus* (K,L) and *Acomys* (M) ears at various time points. Asterisks (K) indicate hair placodes. Arrowhead (L) indicates proliferating chondrocytes. (N-O′) Alcian Blue staining of *Mus* ears at P7 (N) and P9 (O). N′ and O′ show magnifications of the boxed areas in N and O, respectively. Yellow arrowheads indicate maturing cartilage. (P-P″) Neural crest lineage tracing. *Wnt1*^+^ (P; green) structures are neural crest derivatives while *Wnt1^−^* (P′; red) structures are not. Overlay of *Wnt1*^+^, *Wnt1^−^* staining at P11 (P″). (Q) *Wnt1*^+^ elastic cartilage and perichondrium (white dashed line). Scale bars: 100 µm (A-F); 200 µm (G-M); 500 µm (N-O). *n*=2 (G,I-K,M), *n*=3 for all other panels.

We next endeavored to further examine the stages of elastic cartilage differentiation in the ear pinnae of *Mus* and *Acomys* histologically. In embryonic *Mus*, infiltrating lateral tissue was visible, interrupting the central condensation, and by P11 mature vessels were visible within these spaces (Fig. 3A,B). In *Acomys*, a similar sequence of events unfolded such that by E30 maturing vasculature was observed traversing the cartilage (Fig. 3C). These data suggest that foramina locations develop simultaneously with the presumptive cartilage to establish the mature elastic cartilage anatomy.

Next, we investigated the expression of early chondrocyte differentiation markers. BMP signaling regulates chondrogenesis and signaling can be assessed by accumulation of the downstream BMP transcription factor pSMAD1/5/8 (Yang et al., 2024; Yoon and Lyons, 2004). pSMAD1/5/8 was diffusely expressed throughout the mesenchymal tissue at P0, was robustly expressed in the central condensation at P1, and then drastically reduced in all tissues but the muscle by P3 (Fig. 3D-F). SRY-box transcription factor 9 (SOX9), a marker of chondroprogenitors and hair follicle stem cells, was not visible in the central condensation of E20.5 ears, though it was visible in hair follicles (Fig. 3G) (Akiyama et al., 2005; Nowak et al., 2008). At P1, when pSMAD1/5/8 was restricted to the central condensation, SOX9 expression also emerged in the central condensation (Fig. 3H). Together, these data support that cartilage differentiation is initiated at ∼P1 in *Mus*. In *Acomys*, SOX9 was not detectable at E15, but was detectable in the central condensation at E20 (Fig. 3I,J). This is consistent with our findings in *Mus* that SOX9 expression begins before ear unfolding, when hair placodes and skeletal muscle are evident (Fig. 2G,H). We then used KI67, which is expressed by cells in S, G2, and M phases, to investigate cell proliferation (Winking et al., 2004). Interestingly, while KI67 was highly expressed in *Mus* hair follicles and myocytes at P1, little proliferation was observed in the central condensation (Fig. 3K). An increase in proliferation at P5 proximally in the central condensation was observed to coincide with ear unfolding (Fig. 3L). Likewise, in *Acomys* at E20, KI67^+^ cells occurred diffusely throughout the ear, with few positive cells in the central condensation (Fig. S5). At E25, when the ear is unfolding, KI67^+^ cells became visible in the central condensation and, by E30, a high concentration of KI67^+^ cells had accumulated (Fig. 3M). To further visualize maturation of the cartilage matrix we used Alcian Blue, which stains glycosaminoglycans (GAGs). GAGs were abundant in mesenchymal *Mus* tissue at P7, but sparse in the central condensation (Fig. 3N,N′). At P9, a GAG rich matrix was visible forming in the proximal central condensation (Fig. 3O,O′).

Recent work examining mouse embryos up through E18.5 concluded that all tissues in the embryonic ear pinna were of neural crest cell (NCC) origin except for the epidermis and muscle (Minoux et al., 2013; Zhang et al., 2020). To further examine contribution of the neural crest to mature tissue, we tracked NCCs using a widely employed *Wnt1-cre* driver that labels pre-migratory NCCs crossed to *ROSA^mT/mG^* females (Lewis et al., 2013; Muzumdar et al., 2007). In the resulting progeny, cranial NCCs are EGFP^+^/tdTomato^−^, and all other cells are EGFP^−^/tdTomato^+^ (Fig. 3P-Q). At P11, we observed *Wnt1*^+^ cells contributing completely to the elastic cartilage, perichondrium, dermis, dermal papilla and dermal sheath cells (Fig. 3P-Q; Fig. S6A,B). At P50, *Wnt1*^+^ cells also gave rise to adipocytes (Fig. 3P-P″ and Fig. S6C). *Wnt1^−^* cells were observed throughout the skeletal muscle, epidermis, and in most of the hair follicles (Fig. 3P′,P″; Fig. S6A-D). Due to autofluorescence, the hair shaft showed some signal in the GFP channel, but likely is also derived from *Wnt1*^−^ cells. Sparse *Wnt1*^+^ cells associated with the muscle likely represent mesenchymal supporting cells (fascia, vasculature, nerves etc.) or developing adipose (Fig. S6C,D). We also detected a small population of *Wnt1*^−^ cells interspersed throughout the mesenchymal compartment, some of which expressed the pan-macrophage marker IBA1 (Fig. S6E). Other *Wnt1*^−^ cells in the mesenchyme exhibited a fibroblast-like morphology and were likely neural crest-derived cells that did not undergo successful Cre-recombination, as recombination using the *Wnt1-cre* line is <100% (Hari et al., 2012). Taken together, our lineage analysis shows that neural crest-derived cells give rise to elastic cartilage, perichondrium, dermal fibroblasts, dermal papilla, dermal sheath cells and adipocytes in the ear pinna. In contrast, skeletal muscle and epidermal structures in the ear pinna derived from a non-neural crest source. These results provide a detailed developmental origin and maturation process for differentiated cell types of the ear pinna.

### Bmp5 is crucial for elastic cartilage development

**Fig. 4.**
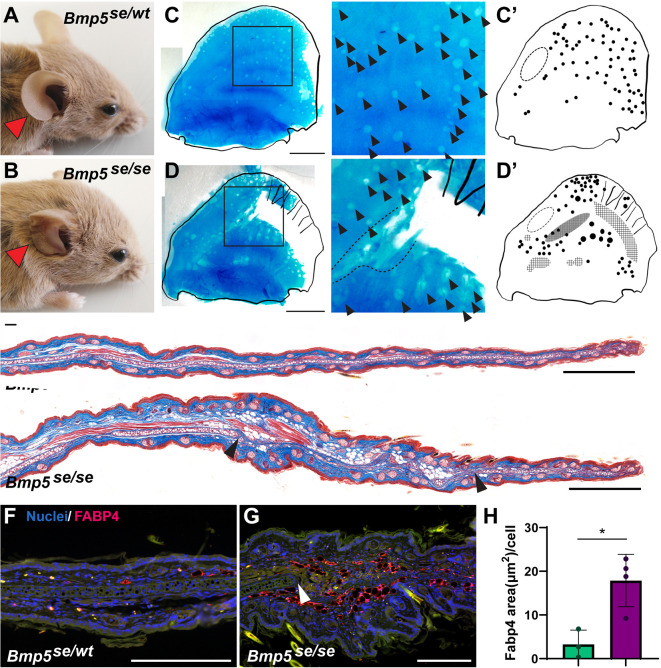
**BMP signaling is crucial for ear pinna cartilage development.** (A,B) *Short-ear* heterozygous (*Bmp5^se/wt^*) *Mus* have grossly normal ears, *short-ear* homozygous mutants (*Bmp5^se/se^*) *Mus* have malformed ears (B). (C-D′) Alcian Blue staining of adult *Bmp5^se/wt^* (C; *n*=3) and *Bmp5^se/se^* (D; *n*=4) ears. Arrowheads indicate foramina. Images on right show magnification of boxed areas on left. (C′,D′) Schematic representation of foramina in the *Bmp5^se/wt^* (C′) and *Bmp5^se/se^* (D′) auricular cartilage. Dashed oval indicates anterior area of uninterrupted cartilage. *Bmp5^se/se^* ears have subjectively larger foramina, with cartilage thinning anteriorly (dotted line, dense crosshatching) and absent posteriorly (crosshatching). (E) Trichrome staining of adult *Bmp5^se/wt^* and *Bmp5^se/se^* siblings (*n*=3 each). Arrowheads show proximal and distal limits of cartilage gap. (F,G) FABP4 immunostaining of the mid-distal ear of *Bmp5^se/wt^* and *Bmp5^se/se^ Mus*. White arrowhead indicates the distal limit of cartilage in *Bmp5^se/se^ Mus*. Green channel is autofluorescence. (H) FABP4^+^ area per cell measured in adult *Bmp5^se/wt^* and *Bmp5^se/se^ Mus*. *P<0.05 (unpaired two-tailed Student's *t*-test). Error bars indicate s.d. Scale bars: 3 mm (C,D); 1 mm (E); 200 µm (F,G).

To extend our cellular analysis of ear pinna development, we sought to investigate cases of abnormal ear development. In the absence of readily available transgenic mutants (ear pinna defects are rarely reported), we investigated the naturally occurring *short ear* mouse strain (Green and Green, 1942). The *short ear* (*Bmp5^se/se^*) mutation (p.R208*) results in the loss of the BMP5 carboxy terminal signaling domain and causes skeletal defects throughout the body, including a malformed ear (Green and Green, 1942; King et al., 1994; Lynch, 1921). A previous histological analysis of *Bmp5^se/se^* animals at P1, P3, and P6 showed that condensing elastic cartilage in the pinna was more diffuse than wild-type ears as early as P3 (Green and Green, 1942). Building on this work, we compared *Bmp5^se/se^* heterozygous mutants (*Bmp5^se/wt^*), whose ears develop normally, with *Bmp5^se/se^* siblings (Fig. 4A,B) and observed that the distal helix appeared to be furrowed and truncated in *Bmp5^se/se^* mice (Fig. 4B).

To characterize cartilage defects, we examined the entire cartilage plate in *Bmp5^se/wt^* and *Bmp5^se/se^* adult mice. *Bmp5^se/wt^* mice had a continuous cartilage sheet like other mouse strains, with foramina more numerous distally as well as an area of uninterrupted cartilage along the anterior edge (Fig. 4C,C′). In *Bmp5^se/se^* ears, the middle to distal (mid-distal) cartilage on the PD axis was thinned (anteriorly) or absent (posteriorly), and several foramina were enlarged (Fig. 4D,D′). Interestingly, *Bmp5^se/se^* mutants retained an anterior uninterrupted area of cartilage. Histologically, both genotypes exhibited normal tissue architecture in the proximal portion and distal tip of the ear pinna (Fig. 4E). However, consistent with our whole mount observations, we found disorganized or missing cartilage and an abundance of adipose cells in the mid-distal *Bmp5^se/se^* ear (Fig. 4E). We examined developing *Bmp5^se/wt^* and *Bmp5^se/se^* ears and found that they were histologically indistinguishable at early time points (P1-P3), with a robust condensation of mesenchymal cells running down the center of the ear (Fig. S7). However, starting at P5 this condensation appeared to be narrower in *Bmp5^se/se^* ears, with cartilage subsequently failing to develop in the middle and distal ear at later stages (Fig. S7).

To identify potential differences in adipose abundance between *Bmp5^se/wt^* and *Bmp5^se/se^* mice, we quantified adipocytes between species using the adipocyte/adipoblast marker, fatty acid binding protein 4 (FABP4) (Fig. 4F,G) (Shan et al., 2013). Using this approach, we found that *Bmp5^se/se^* mice had a significantly greater proportion of FABP4^+^ tissue within the dermis than *Bmp5^se/wt^* mice (Fig. 4H; *P*=0.013). These results suggested that mice harboring two *Bmp5 short ear* mutant alleles might have aberrations in cell differentiation along the chondrogenic and adipogenic lineages.

### *Bmp5^se/se^* mice fail to accumulate chondroprogenitors in the mid-distal ear pinna

**Fig. 5.**
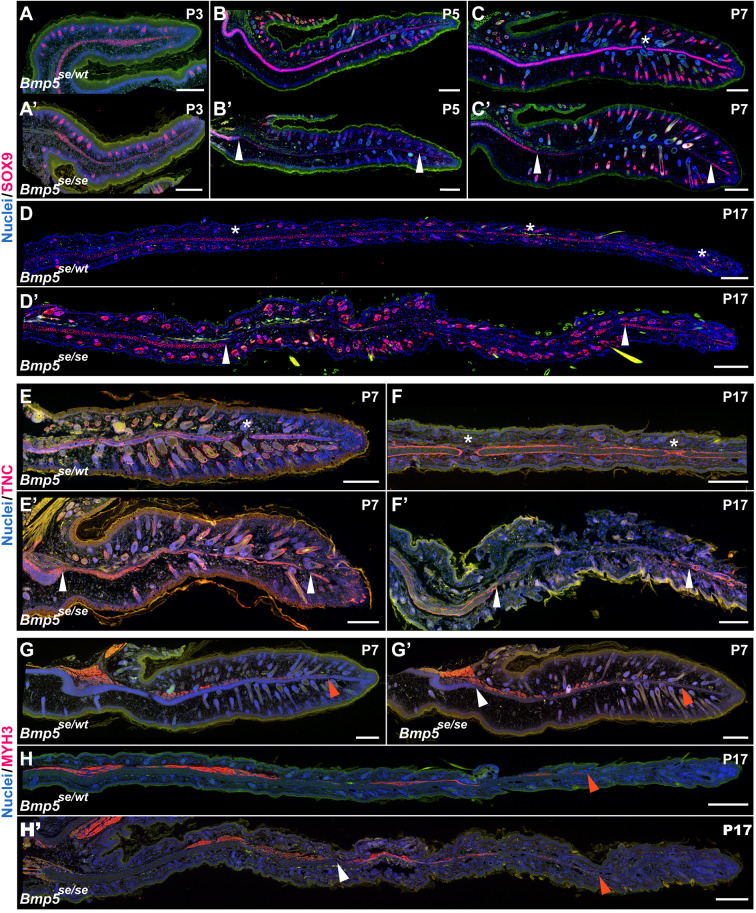
**Cartilage, perichondrium, and muscle development in *Bmp5^se/se^* mouse ears.** (A-H′) Histological comparison of *Bmp5^se/wt^* and *Bmp5^se/se^ Mus* at various developmental time points using SOX9 (A-D), TNC (E-F′) and MYH3 (G-H′) immunostaining. Asterisks indicate foramina in *Bmp5^se/wt^ Mus*. White arrowheads indicate proximal or distal limits of cartilage in *Bmp5^se/se^ Mus*. Red arrowheads indicate distal limit of MYH3^+^ skeletal muscle. Green channel is autofluorescence. *n*=2 (A,B), *n*=3 (C-H). Scale bars: 200 µm.

To further understand the elastic cartilage defects occurring in *Bmp5^se/se^* mice, we examined chondroprogenitors and adjacent tissues. We first examined chondroprogenitor dynamics using SOX9 immunostaining. At P3, before gross histological differences are observable between the central condensation of presumptive cartilage of *Bmp5^se/se^* mutants and their heterozygous siblings, both genotypes expressed SOX9 throughout the central condensation and in developing hair follicles (Fig. 5A), although the SOX9^+^ condensation appeared to be thinned in *Bmp^se/se^* ears (Fig. 5A′). At P5, SOX9^+^ cells densely populated the central condensation of *Bmp^se/wt^* ears (Fig. 5B). *Bmp^se/se^* ears, however, only contained normal SOX9^+^ densities at the proximal and far distal end of the central condensation, with a SOX9 sparse gap spanning most of the ear (Fig. 5B′). Importantly, the dermal papilla expressed SOX9 in both homozygous and heterozygous animals at P5 and P7, and even appeared brighter in homozygous mutants at P17, supporting that reduced SOX9 staining in the developing cartilage was not a technical aberration. Differential SOX9 staining was visible at P7 and P17. *Bmp^se/wt^* ears contained a continuous sheet of SOX9^+^ cells, interrupted only by small foramen at both time points (Fig. 5C,D), while *Bmp^se/se^* ears retained the gap containing rare SOX9^+^ cells through the mid-distal ear pinna at P7 and P17 (Fig. 5C′,D′). The density of hair follicles was similar between *Bmp^se/se^* and *Bmp^se/se^* at P17 and in adult mice (Fig. S8A,B). Serial sectioning of P7 *Bmp^se/wt^* ears showed that the continuous SOX9^+^ central condensation spanned the AP axis of the ear (Fig. S9). Serial sectioning of P7 *Bmp^se/se^* ears showed that the SOX9^−^ gap was largest posteriorly and a continuous central condensation could be observed anteriorly (Fig. S10). Interestingly, in anterior *Bmp^se/se^* ears, the foramina interrupting the central condensation were larger than those observed in *Bmp^se/wt^* ears (Fig. S10). These data suggest that chondroprogenitors are specified but fail to accumulate early in development in *Bmp5^se/se^* ears.

We next sought to determine if perichondrium and muscle, tissues that develop adjacent to the presumptive cartilage, had developmental perturbations in *Bmp^se/se^* ears. During ear development, tenascin C (TNC) localized to the margin of the central condensation, coinciding with the presumptive perichondrium (Fig. 5E,F). We found that TNC nicely demarcated the limits of presumptive cartilage in *Bmp^se/wt^* ears at both P7 and P17 (Fig. 5E,F). However, in *Bmp^se/se^* ears, the TNC^+^ layers appeared to be collapsed against each other, suggesting that the perichondrium had successfully formed, but chondroblasts had failed to differentiate and fill the space between perichondrium layers (Fig. 5E′). These findings are consistent with our trichrome stains showing a thin basophilic presumptive perichondrium with no central condensation in mid-distal P7 *Bmp^se/se^* ears (Fig. S7). At P17, no TNC staining was visible in the cartilage free gap of the *Bmp^se/se^* ear, suggesting that chondrocytes are necessary for maintenance of TNC expression (Fig. 5F′). Interestingly, in *Bmp^se/se^* P7 and P17 ears, muscle appeared to develop normally, extending into the mid-ear even without the presence of a cartilage scaffold (Fig. 5G-H′). These observations provide further insight into how elastic cartilage of the ear pinna develops and how it interacts with the developing perichondrium and muscle.

### Chondroprogenitors in *Bmp5^se/se^* ears fail to expand through proliferation

**Fig. 6.**
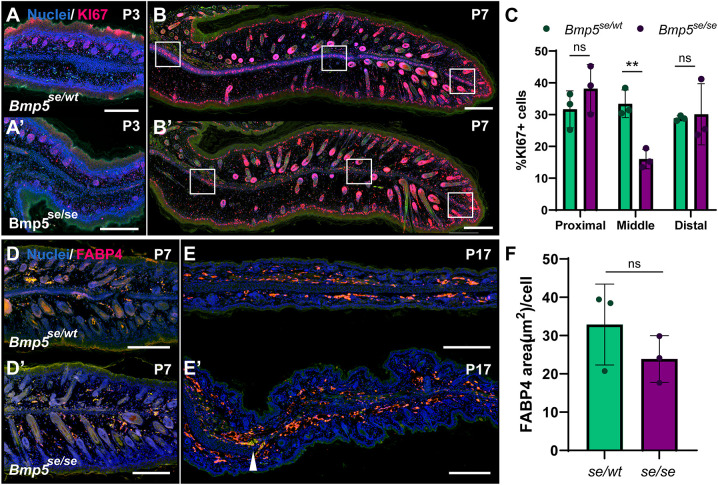
***Bmp5^se/se^* ear pinna chondroprogenitors are deficient in proliferation.** (A-B′) Histological comparison of *Bmp5^se/wt^* (A,B) and *Bmp5^se/se^* (A′,B′) mice using KI67 immunostaining at P3 (A,A′; *n*=2) and P7 (B,B′). (C) Comparison of P7 KI67^+^ cell density within the presumptive cartilage condensation of the proximal, mid, and far distal [white squares (B,B′), 50×50 µm] pinna. (D-E′) Histological comparison of *Bmp5^se/wt^* (D,E) and *Bmp5^se/se^* (D′,E′) mice using FABP4 immunostaining at P7 (D) and P17 (E). White arrowhead indicates proximal start of *Bmp5^se/se^* cartilage gap. Green channel is autofluorescence. (F) Comparison of FABP4^+^ area normalized to cell count in the mid-distal ear pinna at P17. *n*=3 unless otherwise stated. **P<0.01 (unpaired two-tailed Student's *t*-test). Error bars indicate s.d. ns, not significant. Scale bars: 50 µm (A-B′); 200 µm (D-E′).

To determine whether the loss of SOX9^+^ cells in the developing *Bmp5^se/se^* mutant ears resulted from developmental failure or misspecification, we examined proliferation and adipogenesis in *Bmp^se/wt^* and *Bmp^se/se^* ears. BMP signaling is important for both proliferation and differentiation during chondrogenesis (Yoon and Lyons, 2004). Interestingly, a recent study showed that BMPR1 knockdown (KD) in the adult pinna resulted in misspecification of chondroid cells along an osteogenic path, supporting that misspecification underlies some ear defects (Yang et al., 2024). We first marked cycling cells using KI67. Before ear unfolding at P3 and consistent with observations in wild-type *Mus*, *Bmp^se/wt^* and *Bmp^se/se^* ears exhibited KI67^+^ staining in muscle, hair follicles and epidermis, but not in the central condensation (Fig. 6A,A′). At P7, numerous KI67^+^ cells were visible in the central condensation of *Bmp^se/wt^* ears (Fig. 6B). However, *Bmp^se/se^* ears exhibited a gap with few KI67^+^ cells, corresponding to the SOX9 gap (Fig. 6B′). The number of KI67^+^ cells in the far proximal and distal central condensation was similar between genotypes, but there were significantly fewer KI67^+^ cells in the mid-pinna condensation of *Bmp5^se/se^* ears compared to *Bmp5^se/wt^* siblings (Fig. 6C; *P*=0.0048). Tunnel staining of P5 and P7 ears revealed numerous apoptotic cells in hair follicles and epidermis, but not in the central condensation (Fig. S11). These data suggest that chondroprogenitors fail to proliferate in the mid-distal ear of *Bmp^se/se^* mice during postnatal ear pinna development.

Considering the increase in adipocyte abundance we observed in adult mutant ears, and the possibility of an adipochondrocyte progenitor in the ear pinna (Sanzone and Reith, 1976), we investigated whether adipogenesis was increased in the absence of chondroblast expansion. We used FABP4 immunostaining to examine the abundance of adipoprogenitors at P7 and adipocytes at P17. There did not appear to be any difference between *Bmp^se/wt^* and *Bmp5^se/se^* FABP4^+^ cell abundance or distribution at P7 or P17 (Fig. 6D-E′). There was no significant difference in the proportion of FABP4^+^ tissue between *Bmp^se/wt^* and *Bmp5^se/se^* mice at P17 (Fig. 6F). These data support that adipose tissue develops normally in *Bmp5^se/se^*, with an increased abundance of adipocytes occurring post-developmentally in adults. Thus, our data strongly suggest that anatomical defects in *Bmp5^se/se^* ear pinnae arise from failed chondroblast expansion, not differentiation.

### *Bmp5^se/se^ Mus* have impaired chondrogenesis in adulthood

**Fig. 7.**
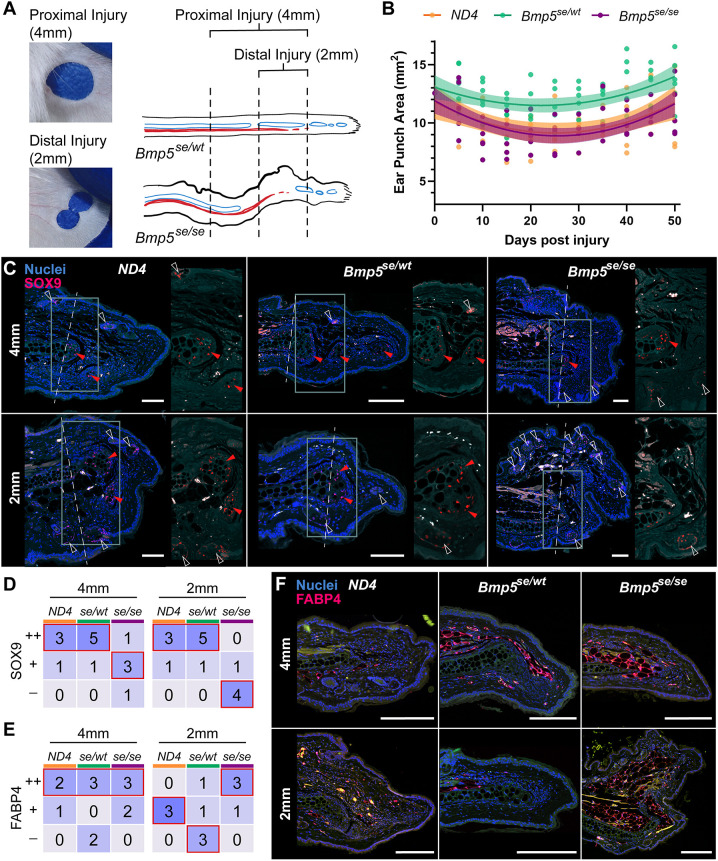
***Bmp5^se/se^ Mus* have impaired chondrogenesis in adulthood.** (A) Representative images and schematic representation of two ear punch wounding paradigms: 4 mm punch (proximal injury) and two 2 mm punches (distal injury). In schematic, blue represents approximate cartilage range and red represents approximate muscle range in the ear pinna. (B) Ear punch area over time following proximal 4 mm ear punch in wild-type (*ND4*; orange, *n*=4), *Bmp^se/wt^* (green, *n*=6), and *Bmp5^se/se^* (plum, *n*=5) mice. Data were collected from three separate experiments. Logarithmic best fit line with 95% confidence. (C) SOX9 immunostaining in scars 50 days post injury. Red arrowheads indicate active SOX9^+^ chondroprogenitors, white arrowheads indicate hair follicles and glands. New cartilage is distinguishable by histology and location. Cyan channel is autofluorescence. Right images show magnification of injury site (dotted line in cyan squares). (D) Number of ND4, *Bmp^se/wt^*, or *Bmp^se/se^* scars 50 days post injury that contained new SOX9^+^ cartilage nodules (++), expansion of SOX9^+^ cells from cartilage end (+), or no SOX9^+^ cells (−). Cells are colored based on the percent of individuals that fell into each category; 100% (dark purple) - 0% (light purple). Red outlined squares indicate most frequent histology of each genotype. (E) Number of *ND4*, *Bmp^se/wt^*, or *Bmp^se/se^* scars 50 days post injury that contained FABP4^+^ cells with lipid vacuoles (++), FABP4^+^ cells (+), or no FABP4^+^ cells (−). Cell colors same as D. (F) FABP4 immunostaining in scars 50 days post injury. Green channel is autofluorescence. Scale bars: 100 µm (C); 200 µm (F).

To determine whether BMP5 is required for chondrogenesis in adulthood, we next challenged *Bmp^se/wt^* and *Bmp5^se/se^* mice with a full thickness injury to the ear pinna. Although *Mus* normally respond to punch injuries with fibrotic repair, they can form cartilage nodules in scar tissue (Gawriluk et al., 2016; Leung et al., 2015). We created 4 mm and 2 mm hole punches in *Bmp^se/wt^* and *Bmp5^se/se^* ears, allowing us to look at chondrogenesis adjacent to the proximal and mid-distal ear cartilage (Fig. 7A). This allowed us to assess injury responses in the normal and abnormal cartilage areas of *Bmp5^se/se^* mice. We measured closure of the proximal 4 mm ear hole over 50 days and compared these to our outbred *ND4* strain, which generates robust scar tissue in response to wounding. Interestingly, we found that 4 mm holes closed similarly in *ND4* and *Bmp5^se/se^* mice, while *Bmp^se/wt^* holes remained significantly larger at multiple time points (Fig. 7B; Fig. S12A,B). Because we could not ensure consistency of the original wound size in distal 2 mm ear punches, we could not statistically assess closure of these injuries (Fig. S12C).

To characterize chondrogenesis in the scar tissue of injured ears 50+ days post injury, we used SOX9 immunostaining. We assessed scars for three potential expression patterns: new SOX9^+^ cartilage nodules within the scar (++), expansion of SOX9^+^ cells from the injured cartilage end into the scar (+), or lack of SOX9^+^ cells in the new scar (−). We found that, following the 4 mm injury, all three genotypes contained SOX9^+^ cells within the new scar tissue (Fig. 7C,D). However, although new cartilage nodules (++) were observable in most *ND4* and *Bmp^se/wt^* ears, most *Bmp^se/se^* ears only had expansion of SOX9^+^ cells at the cartilage end (+), with no additional nodules (Fig. 7C,D). Following the 2 mm wound, while *ND4* and *Bmp^se/wt^* mice were able to generate new cartilage nodules in the scar (++), only one out of five *Bmp^se/se^* mice contained SOX9^+^ cells in their scar tissue (+) (Fig. 7C,D). Positive staining of hair follicle stem cells demonstrated that the SOX9 stain was working in all sections. These data show that *Bmp^se/se^* mice exhibit impaired chondrogenesis into adulthood, even in areas where cartilage develops normally (i.e. proximal region).

To determine if injured adult *Bmp^se/se^* ears also have the concomitant increase in adipose tissue we observed in uninjured ears, we quantified adipocytes in the scar. We assessed scars for three potential expression patterns: mature FABP4^+^ cells with lipid vacuoles in the scar (++), FABP4^+^ cells within the scar (+), no FABP4^+^ cells in the scar (−). We found that all genotypes generated mature fat (++) in scar tissue following a 4 mm injury (Fig. 7E,F). Strikingly, although *ND4* and *Bmp^se/wt^* ears rarely or never generated mature fat following a 2 mm injury, the majority of *Bmp^se/se^* ears contained abundant fat (++) in scar tissue (Fig. 7E,F). These data further corroborated that the local environment of the *Bmp^se/se^* distal ear supports development of adipose tissue.

## DISCUSSION

**Fig. 8.**
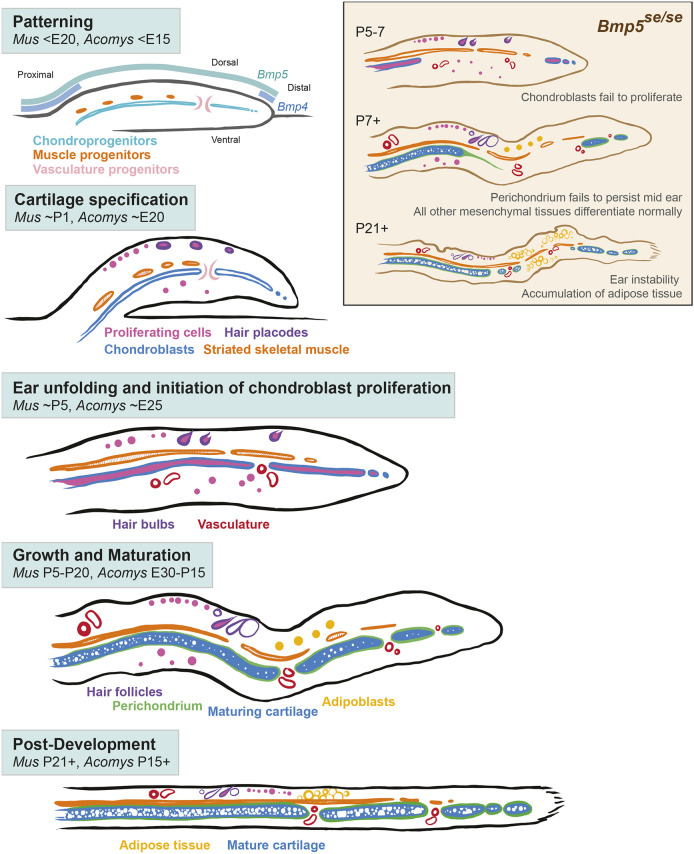
**Maturation of mesenchymal tissues in the rodent ear.** Patterning: the relative pattern of ear pinna tissues is established early in development (*Mus* <E20, *Acomys* <E15). *Bmp5* and *Bmp4* have different expression ranges. The central condensation of chondroprogenitors (light blue) spans the PD axis, interrupted periodically by mesenchyme and presumptive vasculature progenitors (light pink). Muscle progenitors (orange) develop on the dorsal aspect. Cartilage specification: cells of the central condensation are specified into SOX9^+^ chondroblasts (blue) (*Mus* ∼P1, *Acomys* ∼E20). Hair placodes (purple) and striated skeletal muscle (orange) are identifiable. Cell proliferation (pink) is largely confined to the epidermal compartment. Ear unfolding and initiation of chondroblast proliferation: ear unfolding is facilitated by cell proliferation (pink) and muscle (orange) contraction (*Mus* ∼P5, *Acomys* ∼E25). Chondroblasts (blue) begin proliferating and vasculature (red) is identifiable. *Bmp5^se/se^* ears (beige inset) fail to accumulate proliferating chondroblasts in the mid-distal pinna. Growth and Maturation: the ear progressively lengthens on the PD axis and thins on the DV axis (*Mus* P5-20, *Acomys* E25-P15). Differentiated hair follicles (purple) and perichondrium (green) are identifiable. The cartilage matrix (blue) matures. In the absence of cartilage, the perichondrium of the *Bmp5^se/se^* ear fails to persist (beige inset). Post development: the cartilage matrix (blue) matures, and adipose tissue (yellow) accumulates (*Mus* P21+, *Acomys* P15+). Lack of cartilage in the *Bmp5^se/se^* mid-distal ear leads to destabilization in the area of absent cartilage and accumulation of adipose tissue (orange inset).

Here, we established key elements of late gestational and postnatal ear pinna development in two murid rodents: *M. musculus* and *A. dimidiatus*. First, our study revealed a conserved sequence of events leading to formation and integration of skin, elastic cartilage, skeletal muscle, vasculature, nerves, and adipose tissue to form the mature pinna architecture (Fig. 8). Second, lineage tracing demonstrated that connective tissue fibroblasts, elastic cartilage, dermal papilla, dermal sheath cells, and adipocytes in the adult pinna are derived from cranial neural crest. Third, *Bmp5^se/se^* mutant mice revealed that *Bmp5* is necessary for chondroprogenitor expansion in the middle and distal part of the developing cartilage plate; a defect that persists during injury in adult mice. These findings have implications for future development and regeneration studies of the ear pinna including those investigating congenital deformities (e.g. microtia and anotia).

Development of the ear pinna has been described in mice up until E18.5, where it forms as an outgrowth from the side of the head (Fuchs and Tucker, 2015; Minoux et al., 2013). Based on our data, cells condensing within the central region of the developing pinna are elastic cartilage precursors. Before chondrogenesis begins however, craniofacial mesenchyme extends across the anteroposterior axis of the ear, interrupting these condensations (Fig. 8, Patterning). Our detailed analysis indicates that these interruptions give rise to foramina conveying newly forming blood vessels and nerves across the dorsoventral axis of the pinna. Furthermore, our data suggest that vasculogenesis and peripheral innervation coincide with elastic cartilage morphogenesis and that patterning is established well before differentiation. Most tissues in the ear pinna begin differentiating before ear pinna unfolding, except for perichondrium and adipose tissue (Fig. 8, Cartilage specification). Given that unfolding of the ear pinna precedes the onset of skeletal muscle differentiation, contraction of the dorsal myofibers likely contributes to this process along with cell proliferation (Zhang et al., 2020) where chondroblast proliferation contributes to lengthening of the ear (Fig. 8, Ear unfolding and initiation of chondroblast proliferation, Growth and Maturation, and Post-development). These data from *Mus* and *Acomys* support a common developmental plan for the ear pinna and support that *Mus* is a useful proxy for pinna development in other murids.

Previous work has shown that Rhombomere4-derived and Hoxa2-expressing cranial NCCs of the second pharyngeal arch give rise to the embryonic pinna, with the possible exception of the tragus, and that Hoxa2 controls pinna morphogenesis via regulation of Bmp4 and Bmp5 (Minoux et al., 2013). In addition, a neural crest origin of the early pinna, except for epidermis and skeletal muscle, was established (Minoux et al., 2013; Zhang et al., 2020). Using lineage analysis in mice, we confirmed that NCCs are the source of elastic cartilage, perichondrium, dermis, and connective tissue of the ear, whereas the muscle is derived from a different source, consistent with previous studies (Zhang et al., 2020). While some non-NCC derived cells were found throughout the pinna, these may represent infiltrating immune cells. Understanding the origin of cells that give rise to the pinna has significant implications for wound healing, as studies in multiple vertebrate systems have shown that cells replacing damaged tissue exhibit some degree of lineage restriction recapitulating their developmental origins (Kragl et al., 2009; Rinkevich et al., 2011; Rognoni et al., 2016; Singh et al., 2012).

BMP4 and BMP5 have important and complementary functions during ear pinna development, with *Bmp4* expressed primarily at the distal tip and base of the pinna and *Bmp5* expressed throughout the length of the pinna (Minoux et al., 2013) (Fig. 8, Patterning). To explore how dysregulation of BMP signaling might affect the adult ear pinna, we used the previously characterized *Bmp5^se/se^* mouse strain (DiLeone et al., 1998, 2000; Klockars and Rautio, 2009). These mice have a crescent of mis-patterned elastic cartilage with thinning of the cartilage anteriorly and absence of cartilage posteriorly. Our data show that the cartilage phenotype in *Bmp5^se/se^* ears results from a defect in chondroprogenitor proliferation. Specifically, we found that chondroprogenitors form across the PD axis of the *Bmp5^se/se^* ear but fail to proliferate around P5 (Fig. 8, inset). A recent study, which knocked down BMPR1 in auricular chondrocytes of young and adult mice, showed that chondrocytes failed to maintain their identity in the absence of BMP signaling (Yang et al., 2024). Coupled with our data, we conclude that BMP signaling is important for chondrocyte proliferation during early postnatal ear growth and for maintenance of chondrocyte identity in adulthood (Yang et al., 2024). Notably, we observed postural defects developing in older *Bmp5^se/se^* mice that may result from a failure to maintain cartilaginous tissues more broadly. Additional differences between our *Bmp5* mutant and the BMPR1 KD mouse can be attributed to the role of a single BMP ligand (BMP5) compared to all BMPR1-mediated signaling.

Interestingly, a narrow band of cartilage appeared to develop normally at the most distal edge in *Bmp5^se/se^* ears. BMP4 likely compensates for BMP5 but only in the pinna base and distal tip, leading to loss of mid-distal cartilage in the *Bmp5^se/se^* mutant (Antebi et al., 2017). Further studies will be required to determine how different members of the BMP signaling pathway specifically contribute to elastic cartilage formation. The ability of the distal tip cartilage to develop normally is somewhat surprising given evidence that ear pinna cartilage is patterned proximal to distal (Yang et al., 2024), and demonstrates that distal cartilage differentiation is not dependent on proximal cartilage differentiation. Notably, developing skeletal muscle still extended to its normal distal limit. Perichondrium formed across the length of the ear early in development only to disappear later, suggesting that mature cartilage is required to maintain itself. These observations support simultaneous but independent establishment of the different tissue types early in ear pinna development.

Using an ear punch injury model, we found that impaired chondrogenesis within the *Bmp5^se/se^* pinna is retained into adulthood. After injury and subsequent fibrosis, the majority of wild-type and *Bmp5^se/wt^* ear pinna scars contained new cartilage nodules proximally and distally. In contrast, *Bmp5^se/se^* ear pinna scars rarely generated new cartilage nodules proximally and showed almost no chondroprogenitor expansion distally. Apparent inactivity of chondroprogenitors may have resulted from lack of cartilage injury in distal *Bmp5^se/se^* ears. However, even in cases where the 2 mm distal injury crossed cartilage, no chondroprogenitor expansion was observed. As *Bmp5^se/se^* chondroprogenitors have impaired proliferation during development, lack of chondroprogenitor expansion following injury of the adult ear may also result from impaired chondroprogenitor proliferation.

We also observed excess adipose tissue within the dermis of adult *Bmp5^se/se^* mouse ears, especially in areas of missing cartilage. Although some investigators have alluded to an adipochondrocyte progenitor cell, there is little molecular evidence from *in vivo* studies (Sanzone and Reith, 1976). Curiously, *in vitro* stem cell differentiation assays have shown that mesenchymal stem cells are multipotent and can be driven towards both an adipocyte and chondrocyte fate under specific culture conditions (De Bari et al., 2001; Dennis et al., 1999; Gimble and Guilak, 2003; Nöth et al., 2002). We found that there was no increase in adipocytes during ear pinna development in *Bmp5^se/se^* mice and no accumulation of adipose along the midline of the ear, suggesting that chondroprogenitors were not mis-specified. We hypothesize that the lack of mid ear cartilage in the *Bmp5^se/se^* mutants results in a mechanically pro-adipogenic tissue environment (Hossain et al., 2010; Li et al., 2013; Su et al., 2022). Based on our observations, the *Bmp5^se/se^* mouse may be an intriguing model for future studies into the biomechanical control of cell identity.

Distal scars in *Bmp5^se/se^* ears also appeared to favor adipogenesis. It is important to note that FABP4 is also expressed in capillaries, which may have confounded our evaluation. However, mature adipose (++) could be clearly identified by its histological appearance in addition to FABP4^+^ staining, making evaluation of mature adipose reliable. While distal wild-type and *Bmp5^se/wt^* ear pinna scars almost never contained mature adipose, most *Bmp5^se/se^* ear pinna scars contained abundant mature adipose. As in the uninjured ear, we hypothesize increased adipogenesis results from a lack of stabilizing cartilage, creating a pro-adipogenic environment.

Interestingly, *Bmp5^se/se^* animals were able to close more ear punch injury than *Bmp5^se/wt^* animals. Given the more easily deformed anatomy of the *Bmp5^se/se^* ear, contraction likely contributed to improved ear hole closure (Hinz, 2016). In addition, myofibroblast clearance is facilitated by adipogenesis and associated with improved wound healing outcomes (Plikus et al., 2017; Teng et al., 2022). Early contraction followed by myofibroblast clearance through proliferation would be expected to improve healing. Together, our data establish a developmental time course and origin for the tissues of the ear pinna. In addition, they show differential BMP signaling requirements in different parts of the ear pinna elastic cartilage that are retained into adulthood. These data contribute to our broader understanding of craniofacial tissue development and establish a basis for future wound healing and bioengineering strategies.

## MATERIALS AND METHODS

### Animals and tissue collection

Outbred *Mus musculus* (Swiss Webster, Charles River and *ND4*, Envigo; collectively referred to as *ND4*), *Wnt1-Cre* (The Jackson Laboratory, strain 022137; RRID:IMSR_JAX:007676), *ROSA^mT/mG^* (The Jackson Laboratory, strain 007676; RRID:IMSR_JAX:022137), and *SEA/Gn* (The Jackson Laboratory, strain 000644; RRID:IMSR_JAX:000644) animals were maintained in static microisolator cages and *Acomys dimidiatus* were maintained as previously described (Haughton et al., 2016). *Mus musculus* breeding pairs (one male and one to two females of breeding age) were checked for plugs every morning before 9am. Ear pinnae were harvested from mice at E20.5, P0, P1 and every other day afterwards until weaning at P21. *Acomys dimidiatus* breeding cages were set up with one male to three females in large wire mesh cages. As spiny mice do not plug, developmental stage of the embryonic time points was estimated based on embryonic characters including external genitalia, skin, limbs, etc. Tissue samples were collected from embryos estimated to be E15, E20, E25, E30 and E35 and from postnatal time points at P0, P1, P5 and every 5 days after until P25. All collected tissues were fixed overnight at 4°C in 10% neutral buffered formalin (NBF).

To trace neural crest cells *Wnt1-Cre* males were crossed to female *ROSA^mT/mG^* reporter mice. In the *ROSA^mT/mG^* reporter strain all cells express membrane-bound tdTomato unless they undergo a Cre-mediated recombination event, in which case they will lose tdTomato and instead express EGFP. Thus, by crossing *Wnt1-cre* males to *ROSA^mT/mG^* females, all pre-migratory NCCs and their progeny would by EGFP^+^/tdTomato^−^ (Fig. 3P-Q). *Wnt1;ROSA^mT/mG^* pups were fostered to *ND4* mothers to avoid cannibalization. Tissue was collected at P11 when all adult cellular compartments in the pinna had formed, as well as at P50 after the pinna had completely formed. Samples were set in 30% sucrose for 1 h before embedding in OCT (TissueTek, 4583) for cryosectioning without fixation.

The *SEA/Gn* strain segregates the *short ear* allele, a single base point mutation in the *Bmp5* gene. The result of this mutation is a premature stop codon, where the truncated protein lacks the 3′ carboxy signaling motif. *Short ear* mice were maintained by crossing *short ear* heterozygotes (*Bmp5^se/wt^*) to *short ear* homozygous mutants (*Bmp5^se/se^*), resulting in Mendelian litters of heterozygous and null animals. Both breeding strategies (*Bmp5^se/se^* female×*Bmp5^se/wt^* male; *Bmp5^se/wt^* female×*Bmp5^se/se^* male) were used with no apparent difference in breeding efficiency. Tissue was collected from *Bmp*5^+/se^ and *Bmp5^se/se^* sibling offspring at weening and was fixed overnight at 4°C in 10% NBF.

The sex of sampled animals was not assessed due to ambiguous secondary sex characteristics at these pre-weaning ages. All animal procedures were approved by the University of Kentucky Institutional Animal Care and Use Committee (IACUC) under protocol 2019-3254.

### Genotyping

*Short ear Mus* were genotyped phenotypically at weaning. *Bmp5^se/se^* mutant mice have characteristic truncated and malformed ears, while *Bmp5^se/wt^* develop normally. Prenatal *Bmp5^se/wt^* and *Bmp5^se/se^* mice were genotyped using Sanger sequencing according to protocol recommendations by The Jackson Laboratory. DNA was isolated from tail tips using overnight digestion at 55°C in lysis buffer (100 mM Tris-Cl, 5 mM EDTA, 0.2% SDS, and 200 µg/ml Proteinase K), followed by 10 min heat inactivation at 90°C. DNA was PCR amplified to enrich for a *short ear* mutation-containing segment of the *Bmp5* gene using the following primers: F – GAACCATTTCACCAGCTCCT; R – GGAGGCATTACAAAGAGTTTCG. PCR products were purified using gel electrophoresis and gel purification using a QiAquick gel extraction kit (Qiagen, 28704). Purified PCR products were sent for Sanger sequencing using the same primers as used in the PCR amplification step. *Bmp^se/se^* mutants were identified based on the presence of the *short ear* C-to-G substitution.

### Ear pinna injury and measurements

Animals were anesthetized using 3% isoflurane. Ear pinnae were disinfected with a 70% alcohol swab. Full thickness ear punches were made using 2 and 4 mm punch biopsy tools [VWR, 21909-132 (2 mm) and 21909-140 (4 mm)]. Animals were then returned to their cages to recover and monitored over 48 h for discomfort. Ear pinnae injuries were measured every 5 days for 50 days to monitor healing. Ear holes were measured using electronic calipers along the PD and AP axis. Animals were sacrificed before tissue collection. Ears were then removed and fixed overnight at 4°C in 10% NBF.

### Whole mount Alcian Blue staining

Whole ears were harvested from adult individuals and fixed overnight in 10% NBF. Fixed tissues were rinsed three times in phosphate buffered saline (PBS) for 10 min each and then incubated in 2.5% trypsin solution at 37°C for 30 min. Tissues were then washed for 10 min in PBS and the dorsal epidermis, dermis, and muscle layer were peeled away from the underlying cartilage. Partially intact ears were dehydrated by rinsing three times in 70% ethanol for 10 min each and then transferred to 100% ethanol for overnight incubation at 4°C. Dehydrated ears were incubated with 0.2% Alcian Blue solution for 8 h at 25°C, followed by clearing solution (1% KOH/10% glycerol in H_2_O) overnight at 25°C. Tissues were then transferred to storage solution (50% glycerol/50% ethanol) for imaging. Ventral epidermis and dermis were peeled away from the underlying cartilage leaving an intact sheet of cartilage tissue. Cartilage sheets were mounted in storage solution for imaging.

### Histology and immunostaining

Fixed tissue was washed for 10 min in PBS three times and then 70% ethanol three times. Tissue was embedded in paraffin blocks and sectioned at 5 µm for histology and immunostaining. Masson's Trichrome staining was performed using an American Mastertech Kit (KTMTRPT). Alcian Blue staining was performed using a solution of 1% w/v Alcian Blue dissolved in 3% v/v acetic acid and counterstained with Brazilliant (Anantech, NC9982671). Stained histological sections were mounted with XYL seal (Thermo Fisher Scientific, 8312-4). Hair follicle staging during morphogenesis was classified according to published staging guides (Müller-Röver et al., 2001; Paus et al., 1999).

Immunohistochemistry was performed as previously described (Gawriluk et al., 2016). Briefly, tissues were blocked in blocking buffer (PBS +1.5% goat or donkey serum) for 30 min at 25°C. Primary antibodies were diluted in blocking buffer and incubated with tissue overnight at 4°C. Secondary antibodies were diluted in blocking buffer and incubated with tissue for 30 min at 25°C. Antibody details are listed in Table S1. Immunostaining for KI67 was additionally enhanced using biotin-streptavidin amplification, following serum block and before primary antibody – tissue sections were blocked using streptavidin/biotin blocking kit (Vector Biolabs, SP-2002). Immunostaining for IBA1 was carried out using a far red (Cy5 Affinipure) secondary antibody to distinguish positively signal from endogenous tdTomato (See Fig. 3 and Fig. S4). Negative controls were run using an isotyped IgG specific to the primary species. Nuclei were labeled with Hoechst (1:10,000; VWR, 89139-124). Slides were mounted using ProLong Gold (Invitrogen, P36934).

### Image collection and processing

Fluorescent and bright field images were collected using an Olympus BX53 fluorescent deconvolution microscope or Olympus IX83 inverted fluorescent microscope. Brightfield images were taken for whole mount, Masson's trichrome and Alcian Blue. Images of *Wnt1-cre;ROSA^mT/mG^* cryosectioned tissues were obtained using a Leica TCS SP Laser Scanning confocal microscope. Brightness and contrast were optimized using Adobe Photoshop or ImageJ as needed. Fluorescent images were taken in single channels in grayscale for immunostaining. Images were then pseudocolored and brightness, contrast, and histogram maximum/minimum levels were optimized for each channel individually as needed and then overlaid in ImageJ. For all fluorescent images, the 488 nm channel was imaged to control for tissue autofluorescence. Highly auto-fluorescent tissues such as hair shafts and red blood cells appear yellow or white on images.

### Measurements and statistics

Histological characteristics such as total ear thickness and cartilage thickness, as well as nuclei counts and FABP4^+^ areas, were measured using ImageJ software (National Institutes of Health). In Figs 4H and 6F, the FABP4^+^ area was calculated by manually setting a fluorescence threshold for each figure to capture positive staining and using the ‘analyze particles’ function to calculate area. Areas of non-specific staining (i.e. red blood cells) within the particle analysis were manually selected and measured to subtract from the total area and obtain FABP4^+^ area. In Fig. 4D, nuclei were counted using this method, but the image was processed with watershed separation before particle analysis. In Fig. 6F, nuclei were manually counted. For both analyses a 1 mm length of mid ear was processed. In Fig. 6C, cell counts were carried out manually using the ImageJ cell counter plugin in a 50 µm×50 µm area centered on the central condensation.

Gross anatomy measurements were obtained using ImageJ. Adult *Mus* or *Acomys* were ear punched before photographing the ear. A 4 mm ear hole was used to set scale before obtaining PD and AP measurements.

Unpaired two-tailed Student's *t*-tests and one and two-way repeated measures ANOVA and post-hoc analysis were performed using JMP 12 (SAS Institute) or Prism (GraphPad Software). Previous analysis of cell count immunohistochemistry data in ears, using a standard deviation of 0.07, showed that groups of *n*=3 mice were sufficient to detect a species effect with 80% power and α=0.05. For all statistical analyses a minimum of *n*=3 different animals were assessed.

## Supplementary Material



10.1242/develop.204560_sup1 Supplementary information
