# Supplementary material for: Ear pinna growth and differentiation is conserved in murids and requires BMP signaling for chondrocyte proliferation
Source: Development. 2025 Feb 13;152(3):DEV204560. doi: 10.1242/dev.204560 (PMC11883244; doi:10.1242/dev.204560)
Supplement: Supplementary information [file develop-152-204560-s1.pdf]

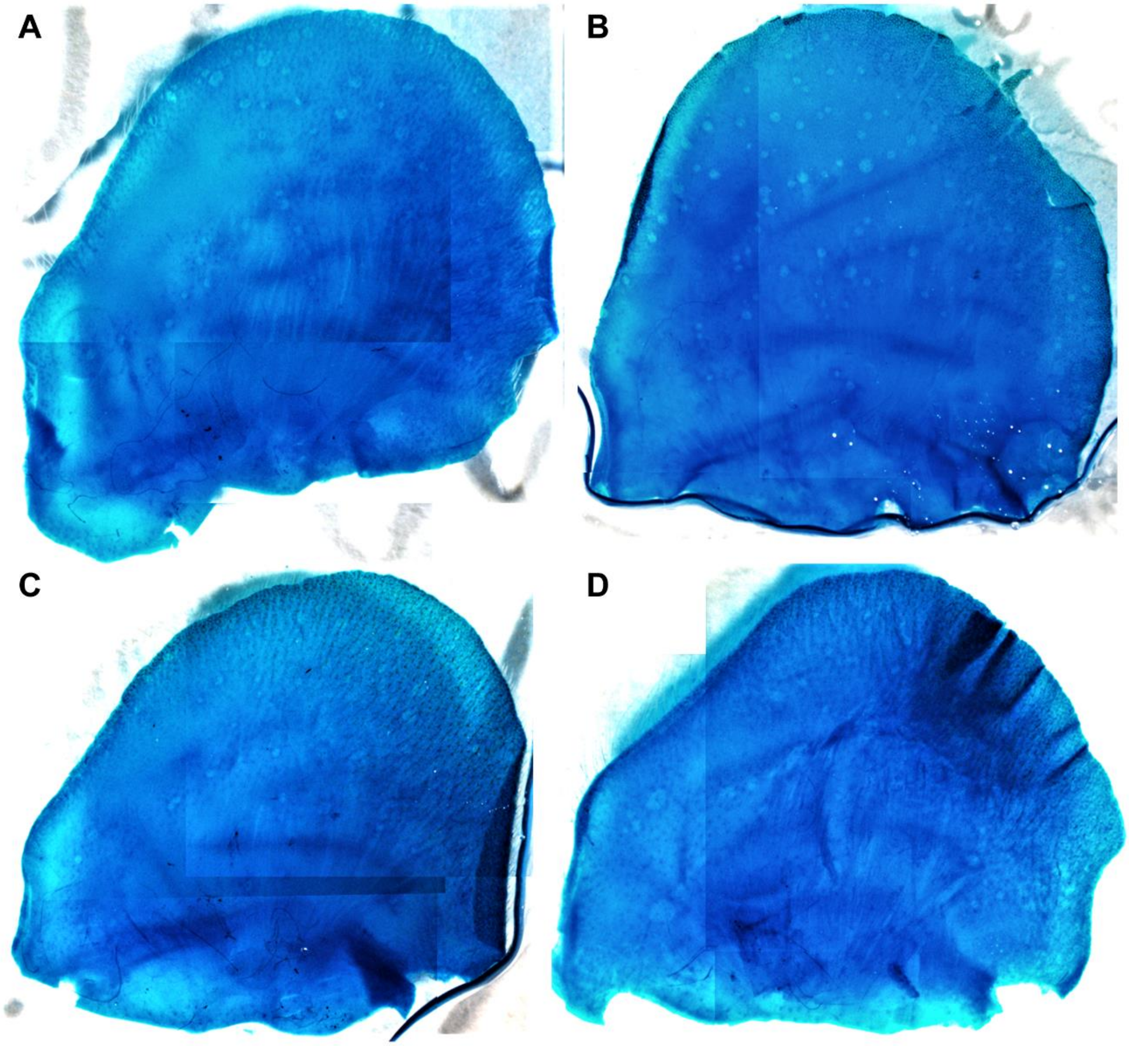

Fig. S1. A-D) Representative images of partially intact adult rodent ears stained with alcian blue. Ears were collected from wildtype (*ND4* or *B6*) *Mus* (A), wildtype *Acomys* (B), *Bmp5*$^{se/wt}$ *Mus* (C), and *Bmp5*$^{se/se}$ *Mus* (D). Dorsal Tissue was removed from all ears prior to alcian blue staining and imaging, leaving cartilage adhered to ventral dermis and epidermis prior to final imaging (Fig. 1 and 4).

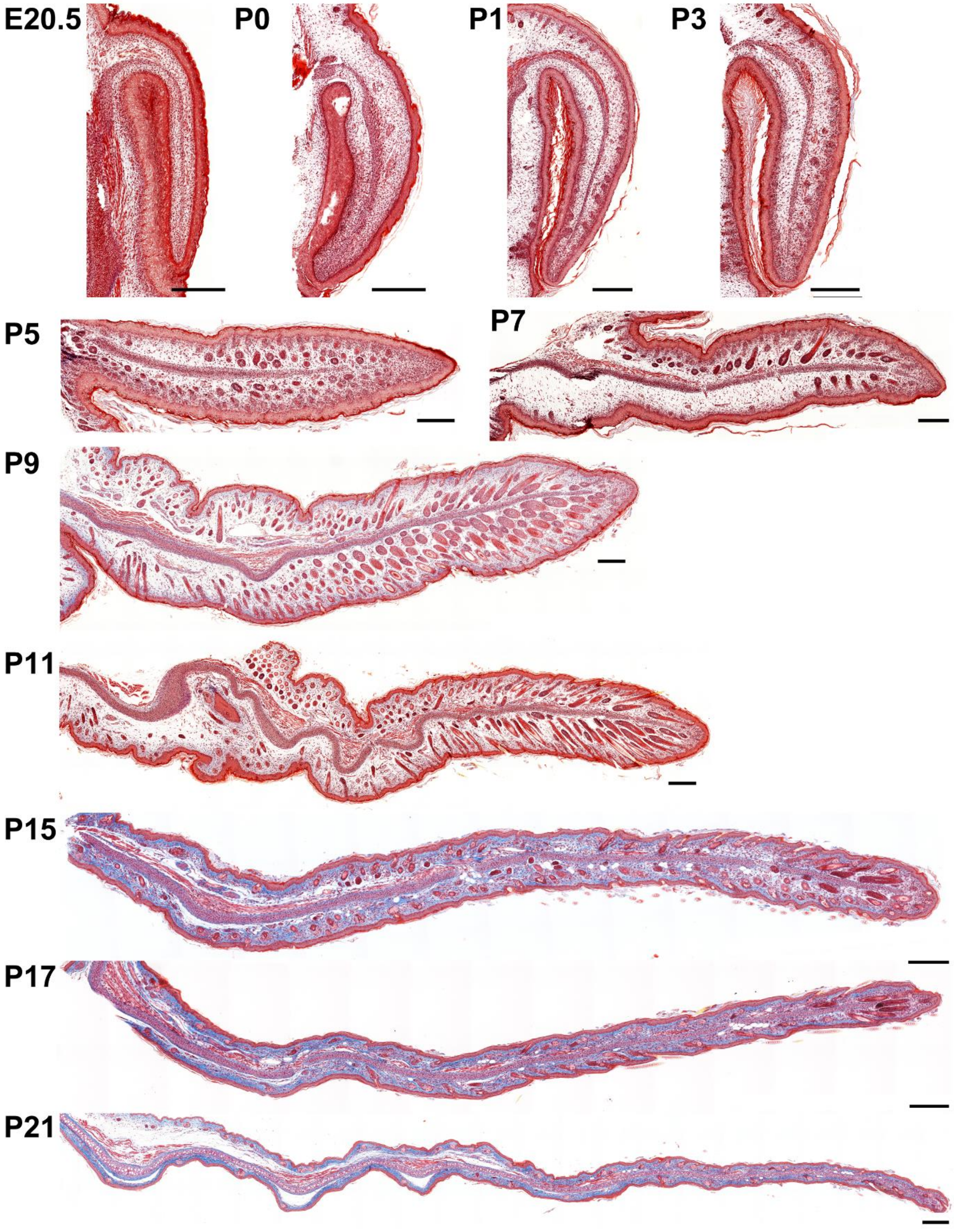

Fig. S2. Representative images of trichrome stained wildtype *ND4 Mus* ear pinnae at various developmental stages. Scale bars = 200µm.

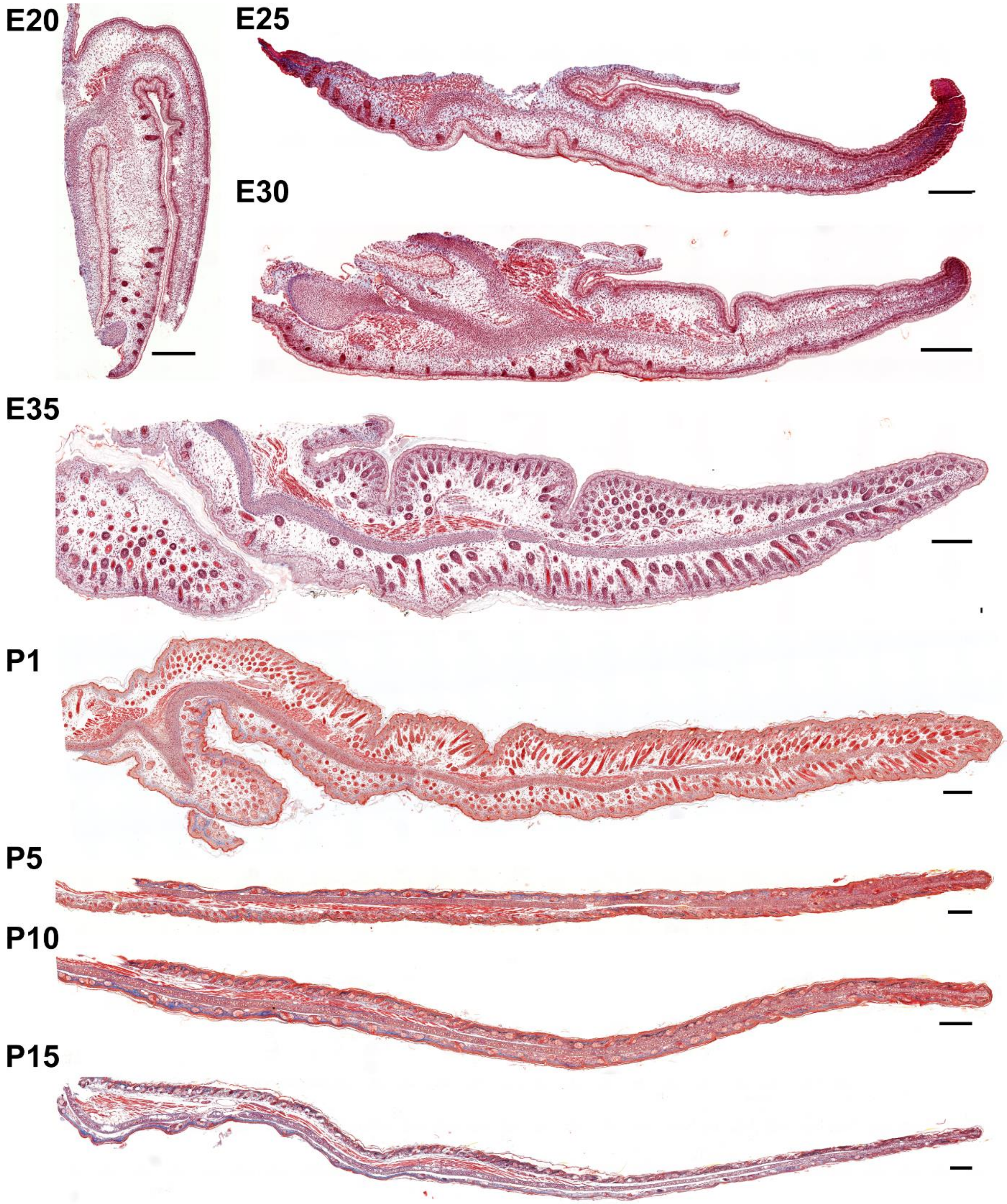

Fig. S3. Representative images of trichrome stained wildtype *Acomys* ear pinnae at various developmental stages. Scale bars = 200µm.

Development • Supplementary information

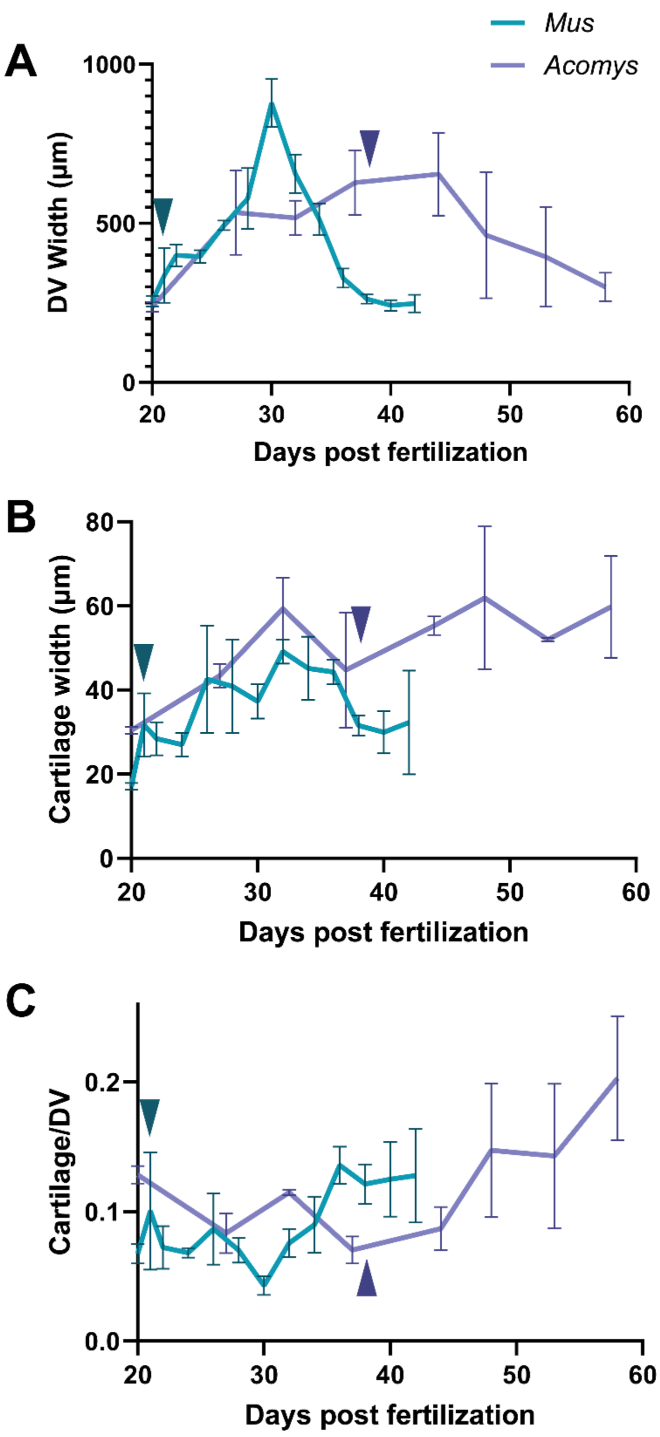

Fig. S4. Dorsoventral and cartilage width measurements from trichrome or SOX9 stained *Mus* (teal) and *Acomys* (purple) ears at different timepoints. Measurements were taken approximately 2/3 down ear shaft from the distal tip, at site where skeletal muscle appearance becomes discontinuous. Arrowheads indicate time of parturition for *Mus* (teal arrow, 21dpf) and *Acomys* (purple arrow, 38dpf). *Mus*; n=3 for E20.5, P1, P3, P5, P11, and P21, n=4 for P15, n=5 for P7 and P9. *Acomys*; n=2 for E25, E30, E35 and P10, n=3 P1, P5, n=4 E20 and P15. Error bars indicate s.d.

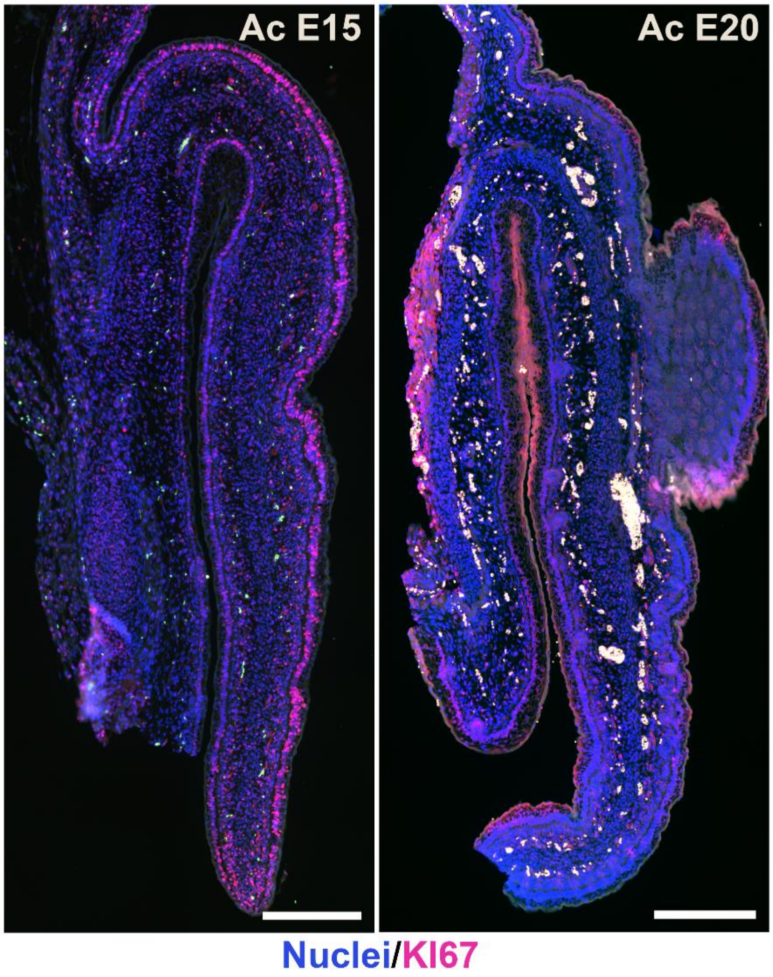

Fig. S5. KI67 Immunostaining of E15 and E20 *Acomys* ears. n=2 for both time points. Scale bars = 200µm. Yellow/green is autofluorescence.

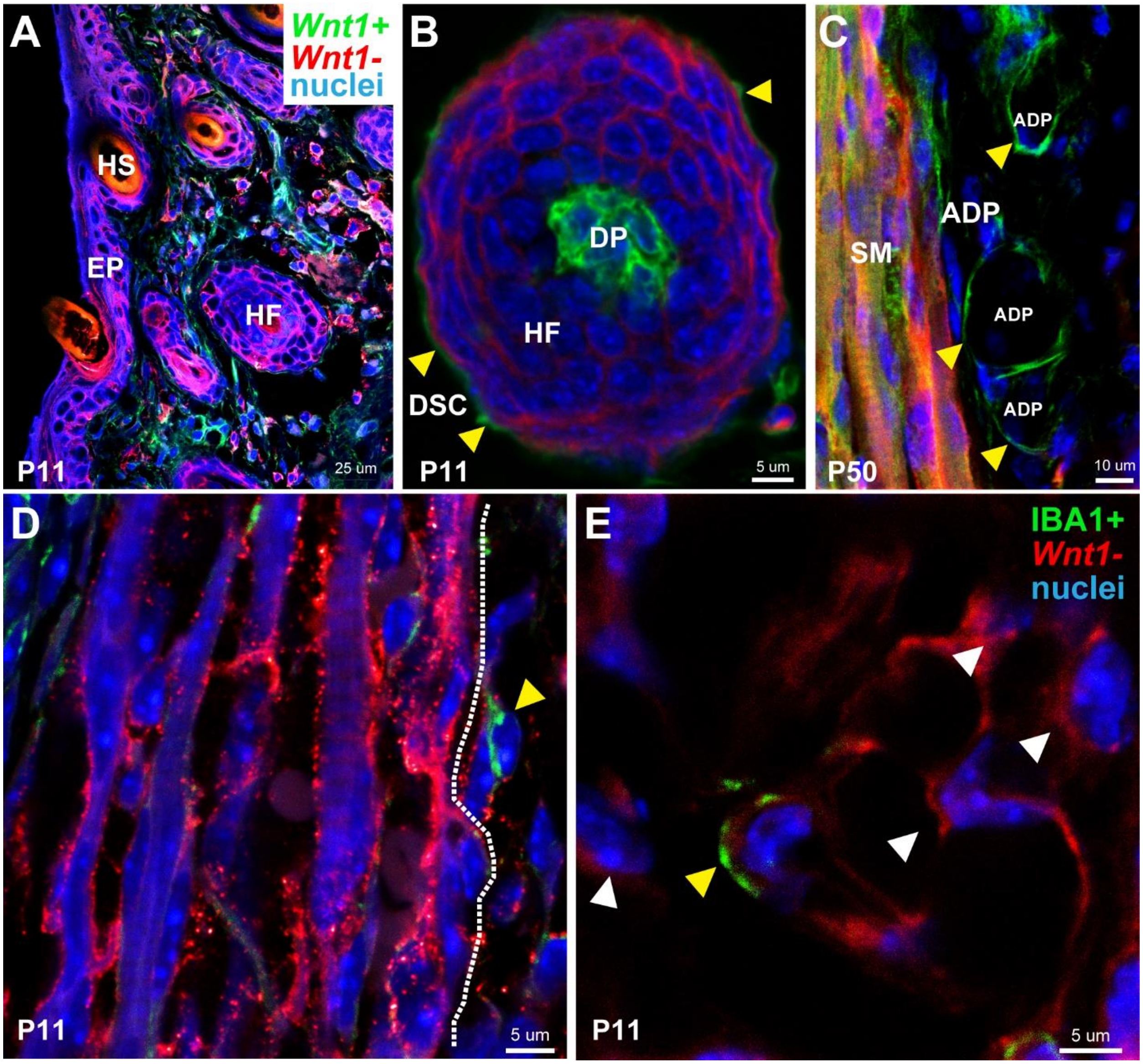

Fig. S6. Neural crest lineage tracing was performed by crossing male *Wnt1-Cre* driver mice to female *ROSA*$^{mT/mG}$ reporter *Mus*. **A-D)** Merged *Wnt1+* (GFP), *Wnt1-* (tdTomato) and nuclei fluorescence. **A)** Epidermis (EP), hair follicles (HF), and hair shafts (ES) are shown. **B)** High magnification of dermal papilla (DP), dermal sheath cells (DSC) and hair follicle (HP). **C-D)** High magnification of adipose (ADP)(C), and skeletal muscle (SM). **E)** Iba1+ immunostained monocytes. Yellow arrows indicate *Wnt*+ (A-D) or IBA1+ (E) cells, white arrows indicate *Wnt*- mesenchymal cells. All panels are P11 ears, except C, which is P50.

Development • Supplementary information

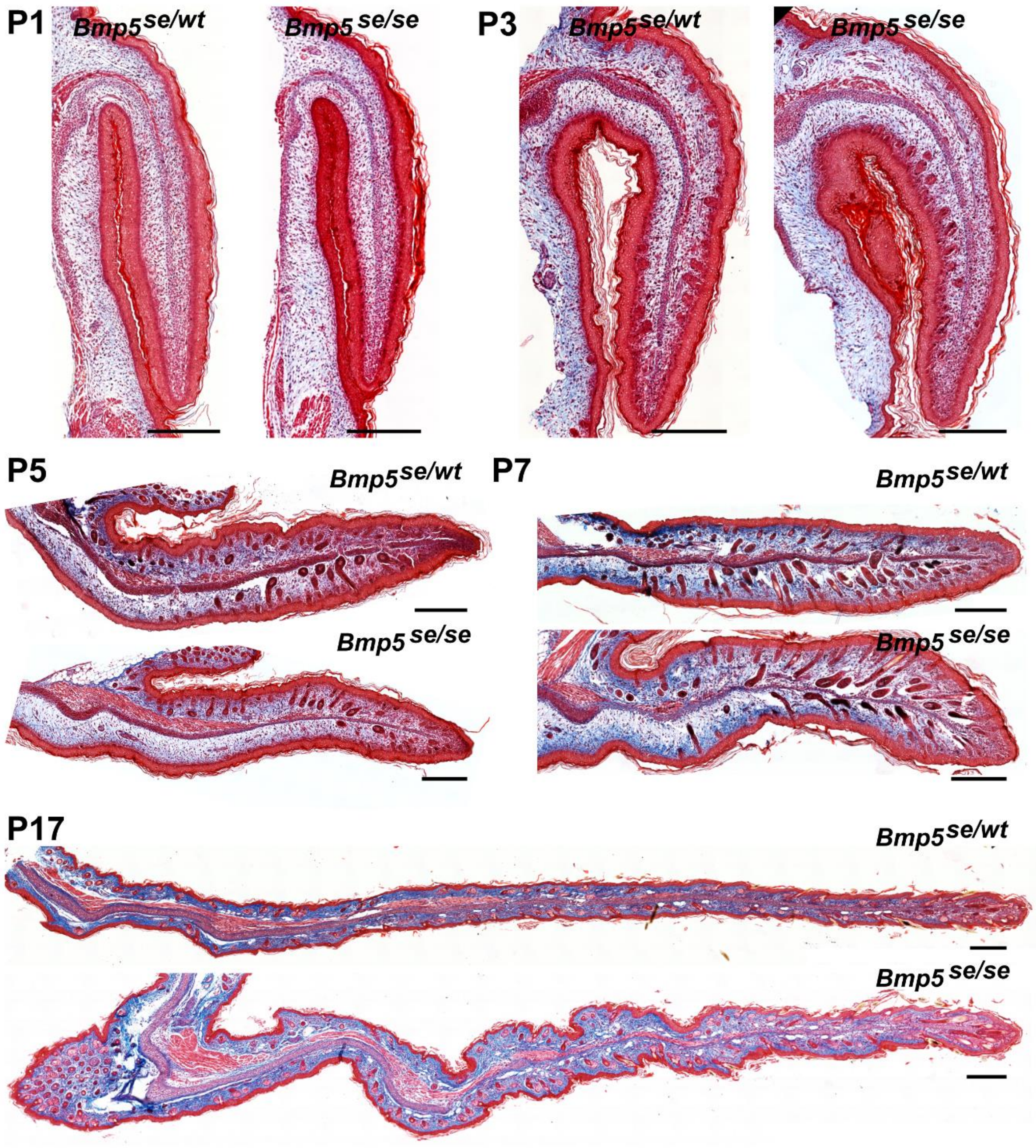

Fig. S7. Representative images of trichrome stained *Bmp5*$^{se/wt}$ and *Bmp5*$^{se/se}$ *Mus* ear pinnae at various developmental stages. Scale bars = 200µm.

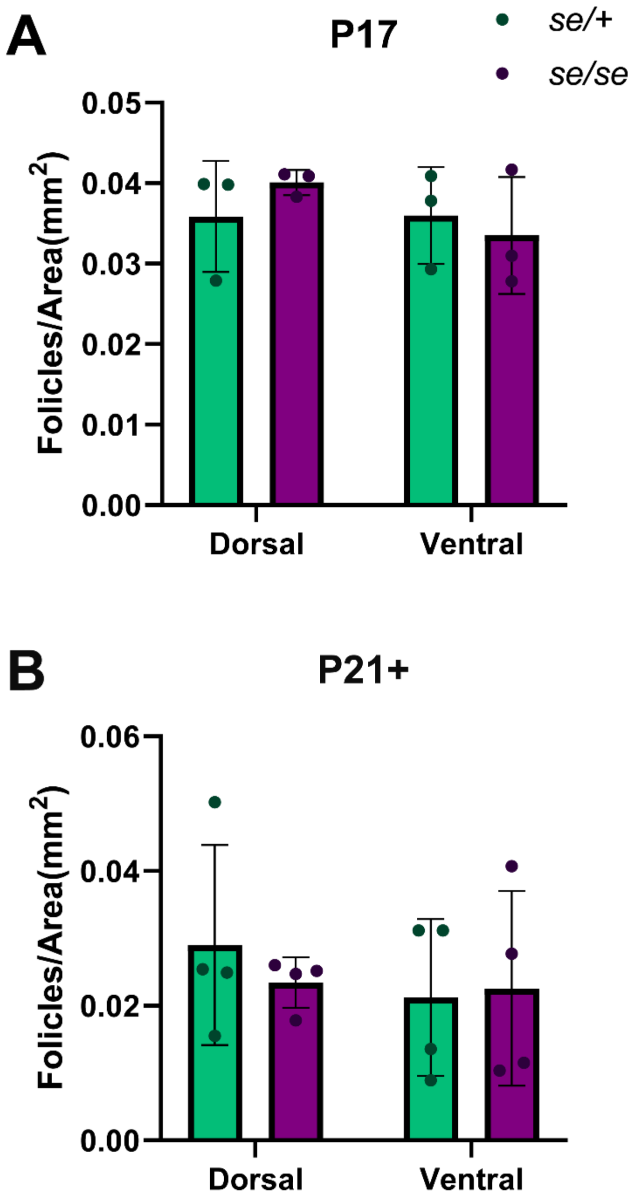

Fig. S8. Hair follicle density in ear pinna skin of P17 and sexually mature (P21+) *Bmp5*$^{se/wt}$ and *Bmp5*$^{se/se}$ *Mus*. Follicles were counted on both the dorsal and ventral aspect of the ear.

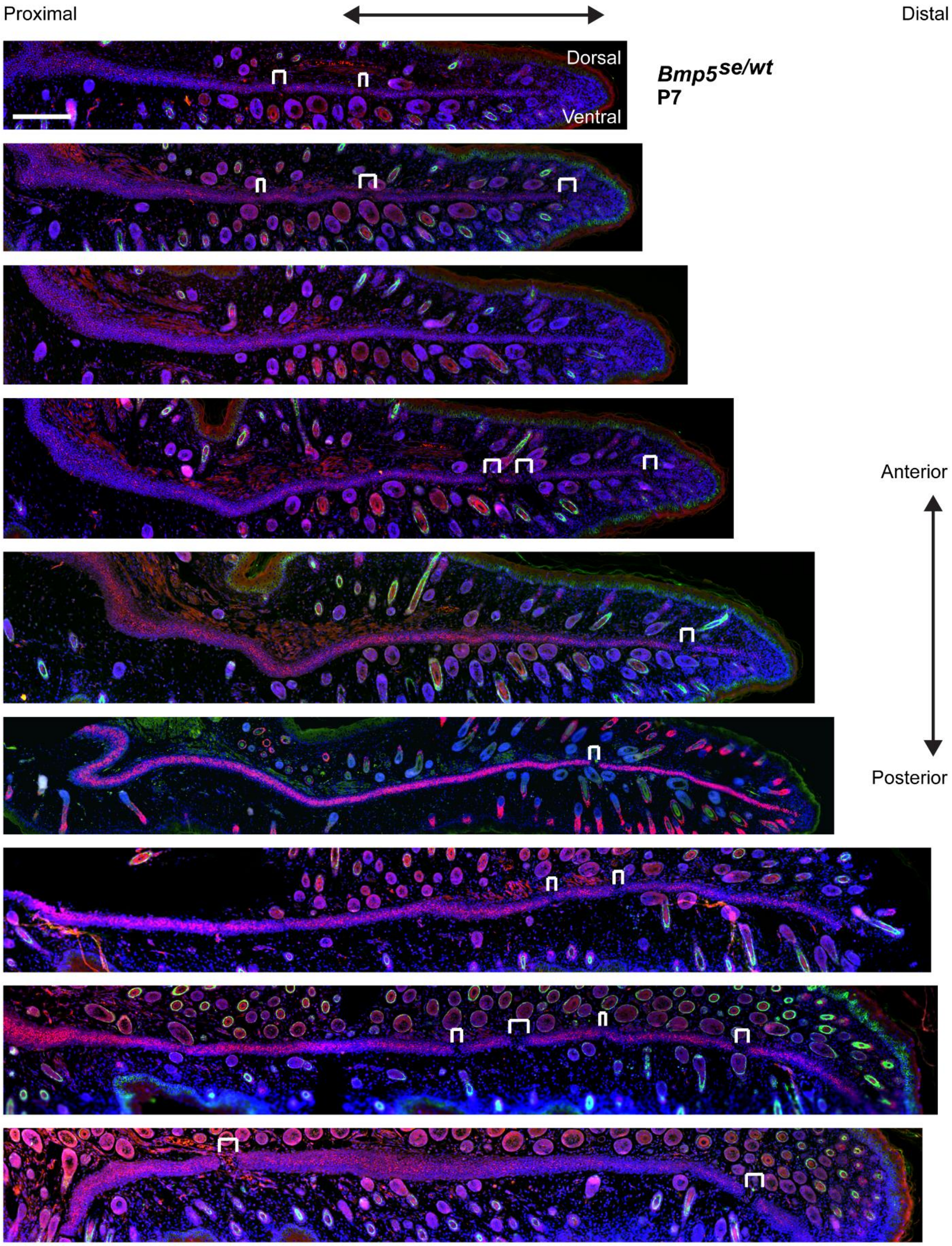

Fig. S9. Serial section of P7 *Bmp5*$^{se/wt}$ ear. Immunostaining of nuclei (blue), SOX9 (red), +/- lectin (green). Brackets indicate interruptions in central cell condensation. Sections taken ~100-300µm apart, ordered anterior (top) to posterior (bottom). Scale bar = 200µm. 6th section taken from Fig 5 and is not stained with lectin.

Development • Supplementary information

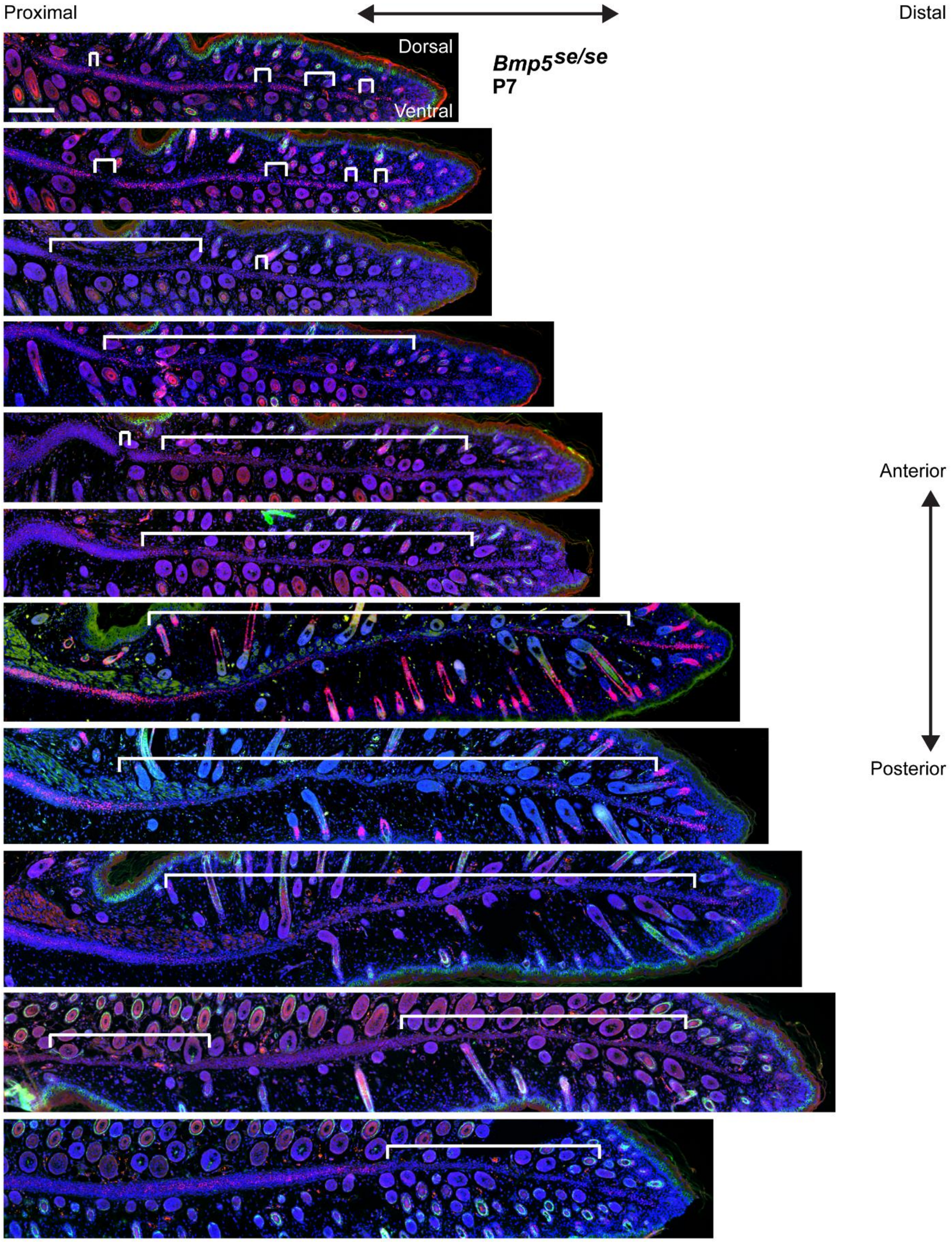

Fig. S10. Serial section of P7 *Bmp5*$^{se/se}$ ear. Immunostaining of nuclei (blue), SOX9 (red), +/- lectin (green). Brackets indicate foramina or absence/thinning of the central cell condensation. Sections taken ~100-300µm apart, ordered anterior (top) to posterior (bottom). Scale bar = 200µm. 7th section taken from Fig 5 and is not stained with lectin.

Development • Supplementary information

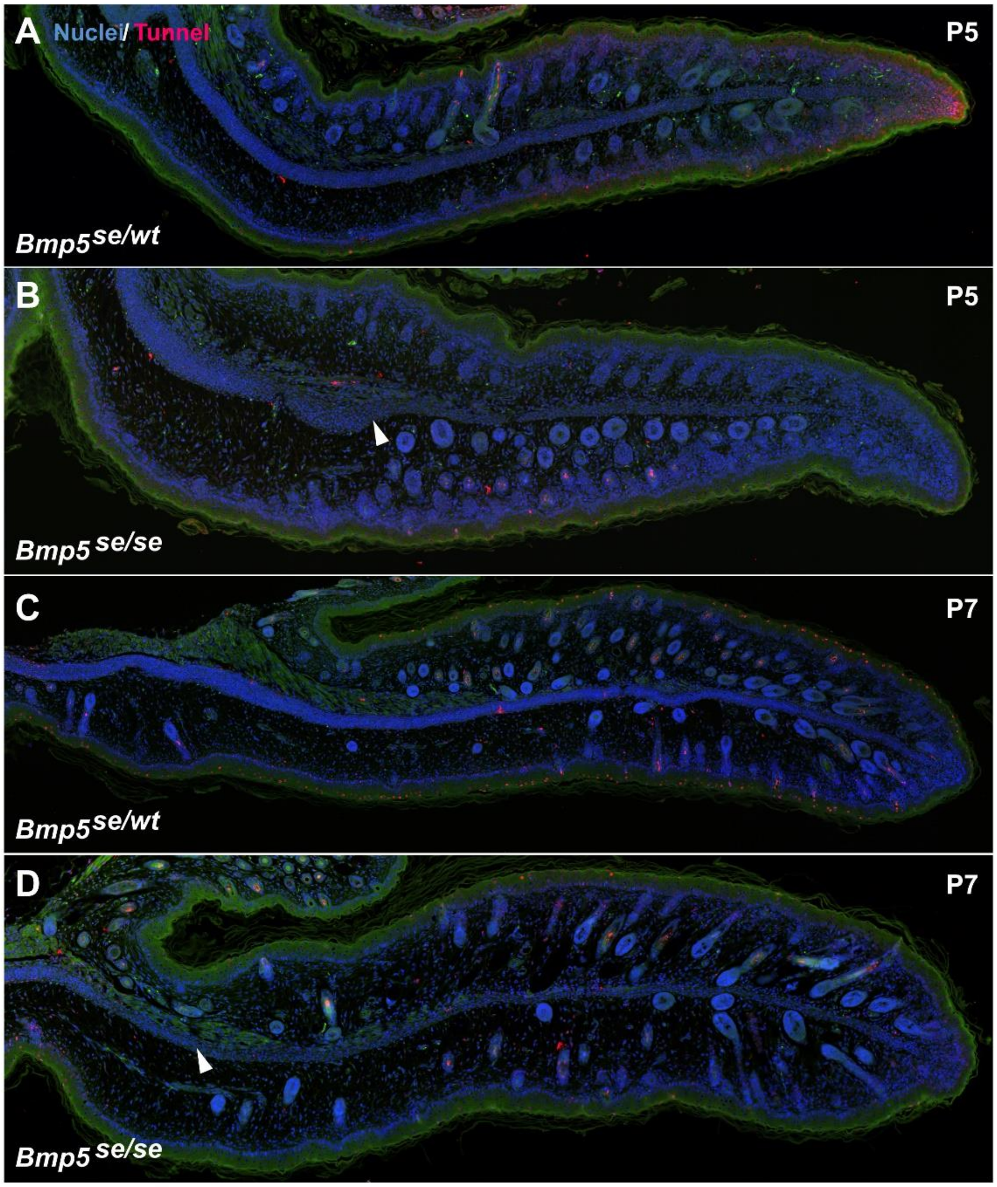

Fig. S11. Tunnel stain of P5 and P7 *Bmp5*$^{se/wt}$ and *Bmp5*$^{se/se}$ ears. Arrowheads indicate distal limit of normal pre-cartilaginous condensation. n=2 for P5 panels, n=3 for P7 panels. Green is autofluorescence.

Development • Supplementary information

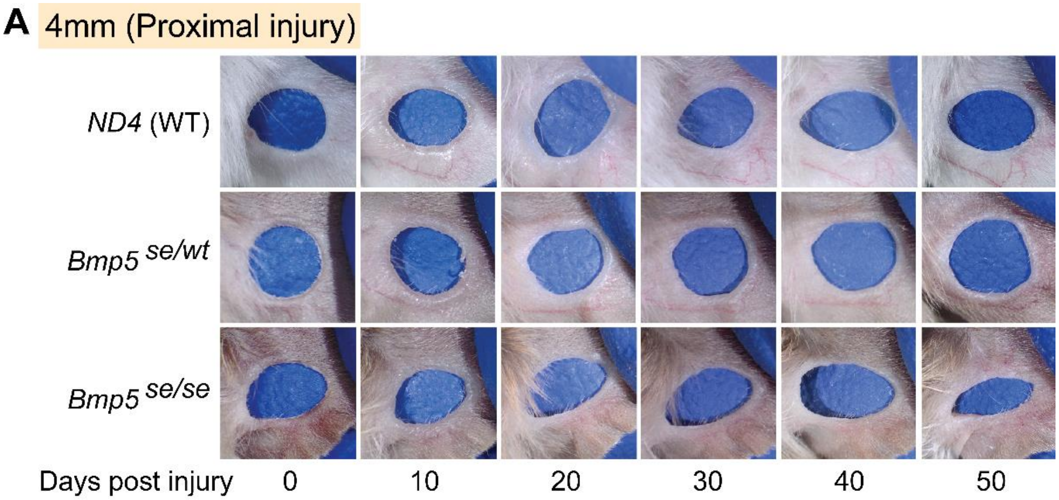

B

	ND4 vs $Bmp5^{se/+}$	ND4 vs $BMP5^{se/se}$	$Bmp5^{se/+}$ vs $Bmp5^{se/se}$
2way repeated measures ANOVA			
Time F value	5.255	9.625	6.416
Genotype F value	6.502	0.02298	12.95
Sidak's multiple comparisons post-hoc test - adjusted P Values			
0 dpi	0.3632	0.1778	0.4557
5 dpi	**0.0476**	0.563	0.1587
10 dpi	0.0614	0.208	**0.0022**
15 dpi	**0.0358**	0.8423	**0.0422**
20 dpi	0.0625	0.9483	**0.0353**
25 dpi	0.1928	0.7624	0.0515
30 dpi	**0.0172**	0.9839	**0.0062**
35 dpi	0.4029	0.7193	0.1989
40 dpi	0.1157	0.7193	**0.0458**
45 dpi	0.1434	0.8925	**0.0077**
50 dpi	0.2189	0.5468	0.093

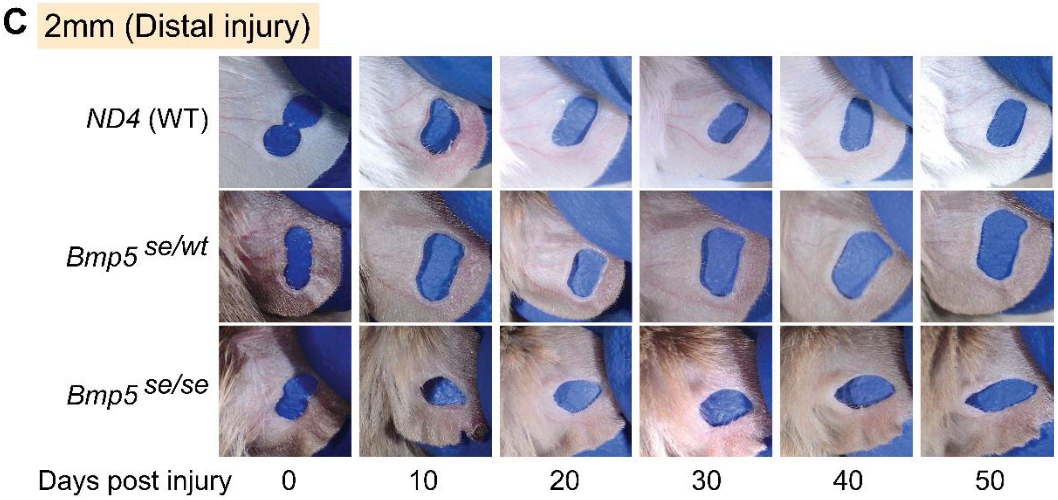

Fig. S12. Ear punch time course and statistics. **A)** Representative ear wounds of wildtype (*ND4*), *Bmp5*$^{se/wt}$, and *Bmp5*$^{se/se}$ *Mus* ears after proximal 4mm full thickness ear punches over 50 days of healing. B) Results of two-factor repeated measures ANOVA analysis on ear hole size following 4mm full thickness ear punch over 50 days. Table shows F value for time and genotype. Sidak's multiple comparison's post-hoc test shows significance values between genotypes at each time point. There was no significant difference in ear hole size between time points within each genotype (i.e. no significant closure of the injury). C) Representative ear wounds of wildtype (*ND4*), *Bmp5*$^{se/wt}$, and *Bmp5*$^{se/se}$ *Mus* ears after distal two 2mm full thickness ear punches over 50 days of healing.

Development • Supplementary information

Table S1. Antibodies used in this study

Primary Antibodies					
Antibody	Company, Cat#	RRID	Host Species	Dilution	Antigen retrieval
anti-Tenascin-C (TNC)	Millipore, AB19013	AB_2256033	Rabbit	1:100	Proteinase K, 2-5min at 25°C
anti-Fatty acid binding protein 4 (FABP4)	Abcam, ab92501	AB_10562486	Rabbit	1:500	heat retrieval in Tris-EDTA ph9 buffer
anti-Myosin heavy chain 3 (MYH3)	Abcam, ab124205	AB_10974234	Rabbit	1:500	None
anti-Ionized calcium binding adapter molecule 1 (IBA1)	Wako, 019-1971	AB_839504	Rabbit	1:1000	None
anti-SRY-box transcription factor 9 (SOX9)	Abcam, ab185230	AB_2715497	Rabbit	1:1000	None
anti-KI67	Abcam, ab15580	AB_443209	Rabbit	1:200	heat retrieval in citrate buffer pH 6

Secondary Antibodies				
Antibody	Company, Cat#	RRID	Host Species	Dilution
anti-rabbit biotinylated IgG	Vector Laboratories, BA-1000	AB_2313606	Goat	1:400
Streptavidin conjugated Alexafluor 594	Life Technologies, 532356	-	-	1:400
Alexafluor 594 anti-Rabbit	Life Technologies, A21207	AB_141637	Donkey	1:400
Cy5 AffiniPure anti-Rabbit	Jackson ImmunoResearch, 711-606-152	AB_2340625	Donkey	1:400

Development • Supplementary information
