# Supplementary material for: Ear pinna growth and differentiation is conserved in murids and requires BMP signaling for chondrocyte proliferation
Source: Development. 2025 Feb 13;152(3):DEV204560. doi: 10.1242/dev.204560 (PMC11883244; doi:10.1242/dev.204560)
Supplement: Supplementary file 1 [file develop-152-204560_review_history.pdf]

# Ear pinna growth and differentiation is conserved in murids and requires BMP signaling for chondrocyte proliferation

Robyn S. Allen, Shishir K. Biswas and Ashley W. Seifert
DOI: 10.1242/dev.204560

**Editor:** Anna-Katerina Hadjantonakis

**Review timeline**

| | |
|---|---|
| Original submission: | 25 October 2023 |
| Editorial decision: | 7 December 2023 |
| First revision received: | 10 September 2024 |
| Editorial decision: | 14 October 2024 |
| Second revision received: | 2 December 2024 |
| Accepted: | 14 January 2025 |


---

**Original submission**

First decision letter

MS ID#: DEVELOP/2023/202474

MS Title: Neural crest cells give rise to non-myogenic mesenchymal tissue in the adult murid ear pinna

Authors: Robyn S Allen, Shishir Biswas, and Ashley W Seifert

I have now received all the referees' reports on the above manuscript, and have reached a decision. The referees' comments are appended below, or you can access them online: please go to BenchPress and click on the 'Manuscripts with Decisions' queue in the Author Area.

As you will see, the referees express considerable interest in your work, but have some significant criticisms and recommend a substantial revision of your manuscript before we can consider publication. If you are able to revise the manuscript along the lines suggested (please also see Editor's note below), which may involve further experiments, I will be happy receive a revised version of the manuscript. Your revised paper will be re-reviewed by one or more of the original referees, and acceptance of your manuscript will depend on your addressing satisfactorily the reviewers' major concerns. Please also note that Development will normally permit only one round of major revision. If it would be helpful, you are welcome to contact us to discuss your revision in greater detail. Please send us a point-by-point response indicating your plans for addressing the referees’ comments, and we will look over this and provide further guidance.

Please attend to all of the reviewers' comments and ensure that you clearly highlight all changes made in the revised manuscript. Please avoid using 'Tracked changes' in Word files as these are lost in PDF conversion. I should be grateful if you would also provide a point-by-point response detailing how you have dealt with the points raised by the reviewers in the 'Response to Reviewers' box. If you do not agree with any of their criticisms or suggestions please explain clearly why this is so.

Editor’s note: The referees recognized that this is a solid paper that presents detailed studies of the pinna development in an outbred mouse and another murid species, in the context of the contribution of neural crest to pinna tissues. The analysis of the short ear mutant mice has offered

© 2025. Published by The Company of Biologists under the terms of the Creative Commons Attribution License (https://creativecommons.org/licenses/by/4.0/).

interesting and important knowledge on pinna development, but further work may be needed to validate the findings which at present might not warrant some of the conclusions.

Reviewer 1 Advance Summary and Potential Significance to Field:

The manuscript by Allen et al, describes a detailed ontogeny of murid ear development in an outbred Mus strain as well as in Acomys. While this first section is descriptive, it is necessary to establish the developmental timeline for the subsequent assessment of the developmental basis of the short ear (se) mutant mouse line, which carries a truncating mutation in Bmp5. This analysis has suggested some intriguing aspects of the development of the pinna that are noteworthy and of potential significance to the field, especially given how understudied pinna development is. Overall, the paper is clearly written and the results generally well presented.

Reviewer 1 Comments for the Author:
A variety of minor issues and some additional concerns are highlighted below.

The utility of comparing two different murid species in terms of their ontogeny, and particularly Acomys given its ability to regenerate it ear tissue without scarring and its longer gestational period, is appreciated. Furthermore, it sets the ontogenic timeframe for the subsequent analyses of the short ear mutant.
However, I am not sure the title truly reflects the more notable findings of the paper, which center more on the basis for the pinna phenotype in the short ear mutant. I would therefore suggest a title that refers to both the ontogeny and the short ear developmental basis if possible.

There are a variety of corrections and edits that are recommended. Most are superficial/minor but a couple may require some consideration regarding presentation of data and their interpretation. Some may benefit from an additional image or two, which may require a little extra work but these should be simple and not time-consuming. These comments/concerns are listed below in the order they appear, not by their significance. The few more significant concerns should be obvious.

In the Introduction:
A couple of statements in the Introduction are either over-simplifications or disregard revisions to the model of early ear development and particularly the contribution of the Hillocks of His. For example, the authors cite (multiple times) a review by Cox et al 2014 when mentioning general observations about ear development. However, they then make comments about the contributions of the Hillocks of His to the final pinna structure, disregarding the recent proposed model in that same review – that is, the Hillocks of His give rise to the proximal structures of the pinnae but that caudal BA2 tissue forms the bulk of the scapha and helix. Furthermore, the model – based on the data of Minoux et al, 2013 – suggest Pharyngeal Arch 1 tissue may in fact give rise to the tragus, contrary to the statement in this manuscript that cites earlier conclusions that only arch 2 tissue contributes to pinna development. While the model proposed in the Cox et al review is just an interpretation of available data, there are no studies that definitively exclude this possibility. So, the authors should be less definitive with their statements.

In the Results section that are a number of errors as well as some data that require clarification.:
1.Line 115. The finding that the pinna is significantly thicker in Acomys than in Mus should be put in context. The pinna, overall, appears larger in Acomys, and so a thicker pinna may be necessary to support the firm or erect appearance of the pinna in adults. In this regard, were Acomys pinnae thicker in the neonatal period as well?
2.Line 165. Suggest adding “as indicated by Fabp4 staining” when referring to adipocyte appearance for the first time.
3.Line 166. Fig 1J should be Fig 2J. Also there is no comparator image to support the statement. I suggest adding a P7 time point to compare with P9. Also it may be valuable to add another image section (or as a Supplementary figure), that better highlights the proximal start of the mature matrix, since the existing Fig 2J does not really show this even though the text implies this is a notable observation.
4.Line 170. Fig 1K should be Fig 2K.
5.Line 220. In relation to Fig 4D (and 4A and 4B), the HS (hair shaft) appears to be both Wnt1- and Wnt+ - does (red/green overlay > yellow coloration).

© 2025. Published by The Company of Biologists under the terms of the Creative Commons Attribution License (https://creativecommons.org/licenses/by/4.0/). 

Does this suggest incomplete excision by Cre? Also, SM seems to be yellow in 4H, suggesting similar – yet this is not mentioned anywhere in the text.
6.Line 224. While the histology is suggestive that the foramen are places where the vasculature innervate/pass through the cartilage, the suggestion that the neural crest cells are forming the vasculature (labeling of a single cell in Fig 4G) needs some validation – perhaps with co-staining for a vascular marker. It should also be considered that there might be some ectopic activity of Cre, hence needing to confirm co-staining of a vascular marker with GFP in more than one area.
7.Line 235. The sentence is incompletely edited. Suggest deleting “using a” at the end.
8.Line 251. This is just a minor observation, and the authors may wish to stay with their current citation and terminology since they are referring to an old publication that uses that particular term. However, it is worth noting that while the earlier studies by Green and Green (1942) refer to the outer pinna as the scapha, the more recent Cox et al (2014) review suggests the outer portion of the mouse pinna may be the equivalent to the human helix, and that there is a visible structure in mouse pinnae that is morphology similar to the scapha in humans (which is a much more limited structure).
9.Line 253 and multiple places on the following pages. Bmp5+/se and Bmp5se/se need to be italicized as these are gene/allele references.
10.Lines 280 and 281. The authors here refer to Bmp5 knockout/null alleles (Bmp5-/- and Bmp5+/-) instead of the short ear allele. This should be corrected.
11.Line 310 and 465. While there may well be two (or three) separate/independently maturing sites of chondrogenesis - which would be a significant finding -, the foramen are abundant and do not separate the cartilage across the ear (Fig 1C & D). So, it is unclear how they demarcate the location of the separation. It may appear that way from the proximal-distal sectioning presented (and associated schematics), but I am not sure this can be concluded from these views. If dorsal-ventral sections/views were assessed, even using alcian blue staining at different ages and this showed that there were indeed distinct site of chondrogenesis, then this conclusion could be drawn. So, the authors either need to do this assessment or remove this statement.
12.Line 366. The authors find that heterozygotes closed significantly less tissue than wildtype and homozygotes but do not clearly state why they believe this is the case. The do mention that the ear punches are more variable because the tissue has less structure in the homozygotes but I am not sure this explains their observation.

In the Discussion section:
13.Line 410. Add “the” before “side of the head”.
14.Line 444. Add “the” before “origin of the early pinna”.
15.Line 452. “complimentary” should be “complementary”.
16.Line 460. Add comma after “midline of the ear” and remove comma from between “ears and “corresponded”.
17.Line 497. This feels as if it should be a new paragraph starting with “Understanding the origin….”
18.Line 501. ‘skin’ is to generic. I suggest using ‘dermis’ instead since the outer layers of the skin are ectodermally derived.
19.Line 504. “notice” should be “noticed”.

In the Materials & Methods section:
20.Line 611. “Hoescht” is mis-spelled. It should be “Hoechst”. Fix elsewhere, including in the various Figure legends as well as the abbreviations used on ALL figures (it should not be Hscht or other variations, but rather Hchst).
In Figure legends:
21.Figure 3 legend – delete “specific” from line 854 as it is used again in the same sentence later on line 855.
22.Figure 4 legend – add “SM” abbreviation and in full as not mentioned but on figure.
23.Figure 7 legend – indicate what the white arrowheads are pointing out (including in the main text).

Reviewer 2 Advance Summary and Potential Significance to Field:
The paper by Allen et al. reads like three separate studies thrown together to make a larger paper. The title sums up a small part of the actual paper and has already been shown so should not be presented as a particularly novel finding. The paper starts with a comparison of ear development in

© 2025. Published by The Company of Biologists under the terms of the Creative Commons Attribution License (https://creativecommons.org/licenses/by/4.0/). 

the spiny mouse and Mus musculus, then moves to confirm neural crest contributions in the pinna, and finally describes the pinna phenotype in the short ear mouse. The last section is more novel but not comprehensive.

Reviewer 2 Comments for the Author:
It is known that the neural crest gives rise to the pinna. A subject of quite a few papers has been the relative contribution of first and second arch crest to the non-myogenic mesenchyme. For example, Minoux showed the contribution of the second arch neural crest to the pinna using Hoxa2 reporter mice (Minoux et al., 2013). They state “the whole mouse pinna derives from Hoxa2 second arch NCCs”.
This was performed using a Wnt1cre Hoxa2 eGFP. This is shown in wholemount and in section. Given this, Wnt1cre mice have been subsequently used to knockout gene expression in the pinna mesenchyme (Zhang et al., 2020).

So that a Wnt1cre reporter labels all the pinna cells, except for the muscle and epithelium, is therefore, completely expected and should be relegated to a supplementary confirmatory image at best. The authors mention that previously the contribution of the pinna was only studied to E18.5, but the pinna is established by this point, even if it continues to grow in size and shape postnatally. The authors mention labelling of blood vessels, which may represent pericytes as these are known to have a neural crest origin in the head. Further markers would be necessary to confirm this.

Given this, the paper is reduced to a descriptive comparison of the pinna in the lab mouse and spiny mouse (Figure 1-3) and an analysis of the short ear mutant (Figure 5-8). The authors show that the short ear mutant has a defect in pinna chondroprogenitor proliferation, and a later increase in adipocytes. They show a correlation between the defect and position of foramen and make some suggestions about elastic cartilage.

They additionally wound the ear and culture pinna cells, which is not mentioned in the abstract.

Figure 1 shows the similarity of the pinna between the lab mouse and spiny mouse.
This is also not mentioned in the abstract. The alcian blue stains indicate holes, but these are not always visible in the histology sections so it would be important to show that they are not an artifact and that the holes in wholemount correspond to gaps for the vasculature. For example, the supplementary images indicate holes for vasculature at P7 but not at P9 or P21, and similarly there are no holes in the later histology of the spiny mouse, so angle of section is clearly important. Its unclear if the holes are created later or the cartilage forms around the vasculature, presumably the latter.
The spiny mouse is very interesting due to its regenerative capacity, but very little extra information is found in the analysis. The comparison of the development of the pinna in both mouse species is nice but descriptive. For example, relative timing of hair follicle and pinna development are shown but its unclear that there is any link.

The paper then examines the phenotype of the short ear mouse which has a mutation in Bmp5. The mice are found to affect a subset of pinna cartilage with more adipocytes. Lineage tracing was not performed so the relationship of these cell types is unclear and the authors hypothesise a role for mechanical force in postnatal accumulation of adipocytes, but this is not tested. The link with defects and foramen location is interesting but rather tenuous. It’s very unclear from Figure 5 whether there is any relationship. Similarly, the presence of perichondrium but no cartilage requires more analysis to strengthen this claim. Fig6G appears to show a gap in tenascin-C.

Overall, this paper needs a major rewrite to focus on the new. The title and abstract need to be completely re written with a focus on the short ear mutant, and more analysis would be required.

Reviewer 3 Advance Summary and Potential Significance to Field:
In this work, Allen and colleagues show that pinna development is largely conserved within murid species and employ lineage tracing experiments to define the origin of all cells within the ear pinna of mice at late gestational and postnatal stages. In addition, they investigate the effect of loss of BMP5 on pinna development and find that the elastic cartilage is likely not formed properly in the distal pinna due to a defect in chondroprogenitor proliferation. This defect persists into adulthood where the regenerative potential of the cartilage in the distal pinna is impaired.

© 2025. Published by The Company of Biologists under the terms of the Creative Commons Attribution License (https://creativecommons.org/licenses/by/4.0/).

This study employs a combination of different histological and immunohistochemistry analysis of ear pinna sections from Mus and Acomys at a range of late gestational and postnatal stages and compares late stages of chondrogenesis in Bmp5+/se versus Bmp5se/se short ear mutant animals. This study is well executed, the data are convincing, and provide novel insights into normal pinna development, especially at postnatal stages, as well as into the role of Bmp5 in pinna chondrogenesis. This study will be a very good reference for the specialists in the field. However, there are several aspects that require additional information and revision before publication to ensure that the advances provided are fully supported by the presented experimental evidence.

Reviewer 3 Comments for the Author:
Major revision issues
1.It is unclear if the pinna is ever fused to the head in Acomys as it is already not fused to the head at E20. Sections showing the fusion at earlier stages would highlight the similarities between ear pinna development in Mus and Acomys.
2.The authors describe that chondrogenesis starts at P3 in Mus (line 158). Chondrogenesis starts with the condensation of mesenchymal cells, which starts already between E12.5 and E14.5 in the pinna of Mus. What the authors observe at P3 may be the onset of differentiation of chondroprogenitor cells into chondrocytes. This should be amended in the text and schematics (Fig. 2O and 3L).
3.The authors describe that Tnc secretion starts at P3 in Mus (line 158), but do not show any data to support this claim. Additional markers for perichondrium and chondroprogenitor differentiation should be tested to prove that chondrocytes in the pinna mature at this stage, such as Col2b, Col9, and Aggrecan. Some of these may also show signal in Acomys pinna in contrast to Tnc.
4.A reference linking TNC expression to perichondrium is missing.
5.Line 186: "…due to precocial development in Acomys, pre-cartilaginous mesenchymal condensation occurred in utero as did the beginning phases of hair follicle morphogenesis (Fig. 3A-F)…" The authors should note that pre-cartilaginous mesenchymal condensation also occurs in utero in Mus (Minoux et al. 2013, Zhang et al. 2020,…)
6.Line 189: "…Of note, unfolding occurred after myogenesis but prior to apparent hair follicle morphogenesis and chondrogenesis, making this the only developmental benchmark that occurs in an order different from Mus…" Please discuss how this statement fits with the findings in Zhang et al. 2020 showing the presence of differentiated muscle cells in the pinna of Mus as early as E14.5.
7.Considering that the expression of BMP4 in the pinna at embryonic stages is located at the base and distal tip of the pinna (Minoux et al. 2013), the authors may discuss the possibility that BMP4 might partially rescue the effect of loss of BMP5.
8.The hypothesis that the foramina correspond to borders of independently maturing areas of chondrogenesis is not strongly supported by the data shown. Does this statement hold true throughout the whole pinna? Do the locations of the sections along the pinna match in BMP5+/se and BMP5se/se animals for the images shown? Do the calculations in panel 6C and 6I take into account the fact that the pinna in BMP5se/se is shorter than in heterozygous animals?
9.EDU tracing experiments or further Ki67 staining at different late gestational and postnatal stages would further support the finding that elastic cartilage does not develop properly due to a proliferation defect of chondroprogenitors in BMP5se/se animals.

Minor points
1.Line 136: "…the ear pinna unfolds during embryonic development in Acomys (Fig. 1F)…" There is no Acomys shown in Fig. 1F. Maybe cite Fig. S2 and S3 here instead of showing different timing of pinna unfolding.
2.Line 148: It is unclear where the stratum corneum, stratum spinosum, and stratum basale are located in Fig. 2A.
3.Line 152: "… At P1, condensing mesenchymal cells continued to form a dense aggregation while the dermal compartment of the pinna expanded resulting in decreased cellular density (Fig. 2B). …"..Fig 2B does not show data of P1 pinna.
4.Line 166: should be Fig. 2J
5.Line 206: "…Previous work inactivating Hoxa2 in mice demonstrated the importance of neural crest migration through the first pharyngeal arch for pinna formation…" It should read: '....second pharyngeal arch for pinna formation.
6.Line 235: part of the sentence is missing.
7.Line 410: typo "analgen" and "from side"
8.Line 440: typo "compared to other body tissues"

© 2025. Published by The Company of Biologists under the terms of the Creative Commons Attribution License (https://creativecommons.org/licenses/by/4.0/).

9.Line 449: "…connective tissue of the ear, whereas the muscle is derived from a different germ layer (likely mesoderm)…" please cite and discuss Zhang et al. 2020, as they have already shown with lineage tracing experiments that muscle cells in the pinna of Mus are not of neural crest origin.
10.Line 590: should be Fig. 4
11.All figures should have colorblind-friendly coloring. Avoid usage of e.g. green and red in the same panel.
12.Immunostaining images of Fig. 5A and D are very dark and thus it is hard to see what the authors want to show.
13.In Fig. 2, 3, S2, and S3 the orientation and location of the sections in particular for embryonic stages should be more clearly specified.
14.In Fig. 2: the legend does not mention what the cyan arrows mean; the legend is missing the annotation for acronyms in panel A,B,C, and F.
15.Fig. 3E and 3F are not mentioned in the text.
16.Fig. 5: errors in the figure legend, e.g. "…Bmp5+/se (E) and Bmp5se/se (F)…" should be (I) and (J)
17.In Fig. 7, what do white arrows point at?
18.Fig. 8: There are two panels labelled E. The figure legend for C and D need to be swapped. Images are very dark and it is hard to see the Sox9 signal.
19.Data shown in Fig. 8B contradicts the statement in the text (line 366).
20.What is the green/yellow signal/channel in immunofluorescent images of Fig. 5, 6, 7, 8?
21.In the author contribution section CKH is mentioned, but s/he is not listed as an author in the first page of the manuscript.

---

**First revision**

<u>Author response to reviewers' comments</u>

We would like to thank the reviewers for their careful consideration of our manuscript and helpful comments. We have refined our comparative analysis to better convey the sequence of developmental processes and tissue differentiation between *Mus* and *Acomys*. The scope of our study expanded in response to reviewer comments to give greater detail into the process of cartilage differentiation. Additionally, we have added data specifically examining the cartilage defects found in the *short ear* and removed some of our more speculative claims. Specific responses to individual reviewer comments are detailed below.

Reviewer 1 Advance Summary and Potential Significance to Field:
The manuscript by Allen et al, describes a detailed ontogeny of murid ear development in an outbred Mus strain as well as in Acomys. While this first section is descriptive, it is necessary to establish the developmental timeline for the subsequent assessment of the developmental basis of the short ear (se) mutant mouse line, which carries a truncating mutation in Bmp5. This analysis has suggested some intriguing aspects of the development of the pinna that are noteworthy and of potential significance to the field, especially given how understudied pinna development is. Overall, the paper is clearly written and the results generally well presented.

Thank you.

Reviewer 1 Comments for the Author:
A variety of minor issues and some additional concerns are highlighted below.

The utility of comparing two different murid species in terms of their ontogeny, and particularly Acomys given its ability to regenerate it ear tissue without scarring and its longer gestational period, is appreciated. Furthermore, it sets the ontogenic timeframe for the subsequent analyses of the short ear mutant. However, I am not sure the title truly reflects the more notable findings of the paper, which center more on the basis for the pinna phenotype in the short ear mutant. I would therefore suggest a title that refers to both the ontogeny and the short ear developmental basis if possible.

© 2025. Published by The Company of Biologists under the terms of the Creative Commons Attribution License (https://creativecommons.org/licenses/by/4.0/).

The title has been revised to reflect the findings in both normal and *short-ear* pinna development. *Neural crest derived chondroprogenitors require BMP5 to proliferate within the* ear pinna

There are a variety of corrections and edits that are recommended. Most are superficial/minor but a couple may require some consideration regarding presentation of data and their interpretation. Some may benefit from an additional image or two, which may require a little extra work but these should be simple and not time-consuming. These comments/concerns are listed below in the order they appear, not by their significance. The few more significant concerns should be obvious.

In the Introduction:
A couple of statements in the Introduction are either over-simplifications or disregard revisions to the model of early ear development and particularly the contribution of the Hillocks of His. For example, the authors cite (multiple times) a review by Cox et al 2014 when mentioning general observations about ear development. However, they then make comments about the contributions of the Hillocks of His to the final pinna structure, disregarding the recent proposed model in that same review – that is, the Hillocks of His give rise to the proximal structures of the pinnae but that caudal BA2 tissue forms the bulk of the scapha and helix. Furthermore, the model – based on the data of Minoux et al, 2013 – suggest Pharyngeal Arch 1 tissue may in fact give rise to the tragus, contrary to the statement in this manuscript that cites earlier conclusions that only arch 2 tissue contributes to pinna development. While the model proposed in the Cox et al review is just an interpretation of available data, there are no studies that definitively exclude this possibility. So, the authors should be less definitive with their statements.

We agree that our introduction did not adequately convey the intricacies of what is known about early ear development and did not dissect competing models for cell contributions to several adult structures. Due to the expanded scope of the paper, the introductory overview of ear pinna development remains simplified. However, the text has been modified to be less definitive.

In the Results section that are a number of errors as well as some data that require clarification.:
1. Line 115. The finding that the pinna is significantly thicker in Acomys than in Mus should be put in context. The pinna, overall, appears larger in Acomys, and so a thicker pinna may be necessary to support the firm or erect appearance of the pinna in adults. In this regard, were Acomys pinnae thicker in the neonatal period as well?

We now include data clarifying these points. Data specifically addressing size differences in adult (Fig. 1) and developing (Fig. S4) *Mus* and *Acomys* has been added to the paper. Pinna thickness through development appears to vary greatly depending on the stage. The increased relative thickness in adult *Acomys* appears to be largely a result of increased amounts of adipose tissue.

2. Line 165. Suggest adding “as indicated by Fabp4 staining” when referring to adipocyte appearance for the first time.

The image referenced has been removed. A reference addressing FABP4 staining in adipoblasts and mature adipocytes has been added in reference to FABP4 staining in Fig. 4F-G (line 262-264)

3. Line 166. Fig 1J should be Fig 2J. Also there is no comparator image to support the statement. I suggest adding a P7 time point to compare with P9. Also it may be valuable to add another image section (or as a Supplementary figure), that better highlights the proximal start of the mature matrix, since the existing Fig 2J does not really show this even though the text implies this is a notable observation.

A P7 picture of alcian blue staining along with pre and post expression stains for other markers has been added to the updated Fig 3. We believe the expanded picture of Fig 3O nicely shows a maturing gag rich matrix in the proximal part of the ear, but not the distal. Figure references have been checked to ensure accuracy and we have corrected the error.

4. Line 170. Fig 1K should be Fig 2K. We have corrected the error.

© 2025. Published by The Company of Biologists under the terms of the Creative Commons Attribution License (https://creativecommons.org/licenses/by/4.0/).

5. Line 220. In relation to Fig 4D (and 4A and 4B), the HS (hair shaft) appears to be both Wnt1- and Wnt+ - does (red/green overlay > yellow coloration). Does this suggest incomplete excision by Cre? Also, SM seems to be yellow in 4H, suggesting similar – yet this is not mentioned anywhere in the text.

These are important points. The hairshaft has some autofluorescence in the 488 and 594 channels, as can be observed in the green channel of other photos in which autofluorescence was used to highlight the tissue architecture. Double staining in the skeletal muscle likely results from interspersed wnt1+ mesenchymal cells (and possibly developing adipose) between muscle fibers, and incomplete cre excision as noted by the reviewer. These considerations have been added to the text (lines 218-222)

6. Line 224. While the histology is suggestive that the foramen are places where the vasculature innervate/pass through the cartilage, the suggestion that the neural crest cells are forming the vasculature (labeling of a single cell in Fig 4G) needs some validation – perhaps with co-staining for a vascular marker. It should also be considered that there might be some ectopic activity of Cre, hence needing to confirm co-staining of a vascular marker with GFP in more than one area.

We agree with the reviewer's point here that more substantial data is necessary to make these claims. Given the sections we have in hand we were unable to definitively confirm or reject our statement. Therefore, specific claims regarding the origin of vascular tissue from neural crest cells have been removed.

7. Line 235. The sentence is incompletely edited. Suggest deleting "using a" at the end.

Edit incorporated. Thank you.

8. Line 251. This is just a minor observation, and the authors may wish to stay with their current citation and terminology since they are referring to an old publication that uses that particular term. However, it is worth noting that while the earlier studies by Green and Green (1942) refer to the outer pinna as the scapha, the more recent Cox et al (2014) review suggests the outer portion of the mouse pinna may be the equivalent to the human helix, and that there is a visible structure in mouse pinnae that is morphology similar to the scapha in humans (which is a much more limited structure).

We have updated to the helix (Line 244), but for the majority of the paper we now refer to this part of the ear as the middle to distal (mid-distal) ear pinna.

9. Line 253 and multiple places on the following pages. Bmp5+/se and Bmp5se/se need to be italicized as these are gene/allele references.

Thank you for pointing this out. There were several places in our manuscript where gene names, that should have been italicized, were mistakenly not. We have corrected these errors throughout the manuscript. *Short-ear* nomenclature has been updated to *Bmp5*$^{se/wt}$ (het siblings) and *Bmp5*$^{se/se}$ (mutants).

10. Lines 280 and 281. The authors here refer to Bmp5 knockout/null alleles (Bmp5-/- and Bmp5+/-) instead of the short ear allele. This should be corrected.
See answer to point #9.

11. Line 310 and 465. While there may well be two (or three) separate/independently maturing sites of chondrogenesis - which would be a significant finding -, the foramen are abundant and do not separate the cartilage across the ear (Fig 1C & D). So, it is unclear how they demarcate the location of the separation. It may appear that way from the proximal-distal sectioning presented (and associated schematics), but I am not sure this can be concluded from these views. If dorsal-ventral sections/views were assessed, even using alcian blue staining at different ages and this showed that there were indeed distinct site of chondrogenesis, then this conclusion could be drawn. So, the authors either need to do this assessment or remove this statement.

© 2025. Published by The Company of Biologists under the terms of the Creative Commons Attribution License (https://creativecommons.org/licenses/by/4.0/). 

We very much appreciate this comment which caused us to revisit this point in much greater detail and generate additional data. It seems that by pure chance we obtained heterozygous sections from multiple animals with similar foramina distribution. Serial sections of P7 a *short ear* mouse ear (Fig. S8) and het sibling (Fig. S7) have been added to the supplemental to show how foramina locations are scattered across the ear cartilage. Observations about the distal and proximal limits of foramina have been removed from the text.
Furrows in the *short ear* mouse ears make DV sectioning very difficult to obtain the cartilage range in a single or small set of sections, especially considering the narrow DV width of the cartilage. We attempted whole mount alcian blue staining, in developing ears but the strong staining in the mesenchyme prevented us from evaluating the cartilage. We also attempted whole mount SOX9 immunostaining, but were hindered by skin autofluorescence and did not have time to develop the technique. We believe this is an important future direction to further assess the order of cartilage maturation.

12. Line 366. The authors find that heterozygotes closed significantly less tissue than wildtype and homozygotes but do not clearly state why they believe this is the case. The do mention that the ear punches are more variable because the tissue has less structure in the homozygotes but I am not sure this explains their observation.

We appreciate the request for us to provide a hypothesis based on the data we present in the manuscript. Our data is now considered in the discussion (lines 492-497). We believe improved closure may have resulted from a combination of contracture in the absence of cartilage resistance and a pro-adipogenic environment allowing myofibroblast clearance. Of course, a follow-up study would be required to find additional support for this hypothesis.

In the Discussion section:
13. Line 410. Add “the” before “side of the head”.

Added (line 391)

14. Line 444. Add “the” before “origin of the early pinna”. Line modified (line 412)

15. Line 452. “complimentary” should be “complementary”. Corrected (line 424)

16. Line 460. Add comma after “midline of the ear” and remove comma from between “ears and “corresponded”.
Sentence has been removed

17. Line 497. This feels as if it should be a new paragraph starting with “Understanding the origin….”
Line has been moved (line 419)

18. Line 501. ‘skin’ is to generic. I suggest using ‘dermis’ instead since the outer layers of the skin are ectodermally derived.
Line removed

19. Line 504. “notice” should be “noticed”. Line removed

In the Materials & Methods section:
20. Line 611. “Hoescht” is mis-spelled. It should be “Hoechst”. Fix elsewhere, including in the various Figure legends as well as the abbreviations used on ALL figures (it should not be Hscht or other variations, but rather Hchst).
The spelling in the methods section has been corrected (line 595). Figures have been updated to read “nuclei” in place of “Hscht”.

In Figure legends:
21. Figure 3 legend – delete “specific” from line 854 as it is used again in the same sentence later on line 855.
Line removed

© 2025. Published by The Company of Biologists under the terms of the Creative Commons Attribution License (https://creativecommons.org/licenses/by/4.0/).

22. Figure 4 legend – add "SM" abbreviation and in full as not mentioned but on figure.
Abbreviation specified. Data now in Fig S5.

23. Figure 7 legend – indicate what the white arrowheads are pointing out (including in the main text).
Added

***** Reviewer 2 Advance Summary and Potential Significance to Field:
The paper by Allen et al. reads like three separate studies thrown together to make a larger paper.
The title sums up a small part of the actual paper and has already been shown so should not be presented as a particularly novel finding. The paper starts with a comparison of ear development in the spiny mouse and Mus musculus, then moves to confirm neural crest contributions in the pinna, and finally describes the pinna phenotype in the short ear mouse. The last section is more novel but not comprehensive.

Reviewer 2 Comments for the Author:
It is known that the neural crest gives rise to the pinna. A subject of quite a few papers has been the relative contribution of first and second arch crest to the non-myogenic mesenchyme. For example, Minoux showed the contribution of the second arch neural crest to the pinna using Hoxa2 reporter mice (Minoux et al., 2013). They state "the whole mouse pinna derives from Hoxa2 second arch NCCs". This was performed using a Wnt1cre Hoxa2 eGFP. This is shown in wholemount and in section. Given this, Wnt1cre mice have been subsequently used to knockout gene expression in the pinna mesenchyme (Zhang et al., 2020).

So that a Wnt1cre reporter labels all the pinna cells, except for the muscle and epithelium, is therefore, completely expected and should be relegated to a supplementary confirmatory image at best. The authors mention that previously the contribution of the pinna was only studied to E18.5, but the pinna is established by this point, even if it continues to grow in size and shape postnatally. The authors mention labelling of blood vessels, which may represent pericytes as these are known to have a neural crest origin in the head. Further markers would be necessary to confirm this.

We agree that the early stages of ear development have been addressed by numerous studies which we cite in our manuscript. To our knowledge, however, there have been no studies to date that have determined the origin of individual tissues within the adult ear pinna. Having said that, the reviewer will now appreciate that this finding does in fact occupy a relatively small part of the manuscript. We agree that based on cell lineage in other cranio-facial tissues, our findings are not surprising. We have moved a large portion of this data to the supplement and have removed discussion of the origin of vasculature. The paper title has been modified to reflect the full scope of the paper. (And improved cohesion of the data)

Given this, the paper is reduced to a descriptive comparison of the pinna in the lab mouse and spiny mouse (Figure 1-3) and an analysis of the short ear mutant (Figure 5-8). The authors show that the short ear mutant has a defect in pinna chondroprogenitor proliferation, and a later increase in adipocytes. They show a correlation between the defect and position of foramen and make some suggestions about elastic cartilage.

We hope the reviewer will agree that the scope of the paper has expanded to address their comments and the fair amount of new data has increased the depth of our study. The cross species comparison reveals a conserved set of processes that produce the final structures of the ear pinna, including timing relative to parturition. Specifically, our paper examines the sequence of cartilage specification, growth, and differentiation to provide a firm foundation for interpreting pinnae defects in the *short ear* mutant. In addition, we further examine development of adjacent tissues in the *short ear*. Moreover, our discovery that the elastic cartilage does not form a continuous sheet but instead is interrupted by foramina that form early during cartilage differentiation is completely novel. This actually helps explains the discontinuous nature of regenerated elastic cartilage in spiny mice.

© 2025. Published by The Company of Biologists under the terms of the Creative Commons Attribution License (https://creativecommons.org/licenses/by/4.0/).

They additionally wound the ear and culture pinna cells, which is not mentioned in the abstract.
This experiment is now mentioned in the abstract.

Figure 1 shows the similarity of the pinna between the lab mouse and spiny mouse. This is also not mentioned in the abstract. The alcian blue stains indicate holes, but these are not always visible in the histology sections so it would be important to show that they are not an artifact and that the holes in wholemount correspond to gaps for the vasculature. For example, the supplementary images indicate holes for vasculature at P7 but not at P9 or P21, and similarly there are no holes in the later histology of the spiny mouse, so angle of section is clearly important. Its unclear if the holes are created later or the cartilage forms around the vasculature, presumably the latter.

As adult anatomical similarities have been noted in other studies, we have opted not to emphasize this data in the abstract. Histology of the cartilage foramen has been added to Fig. 1G-H to highlight vasculature and nerves passing through them and these are clearly visible in the foramina. Similar structures are visible across the developmental series in Fig. S2, S3, and S6. Due to the distribution of foramen throughout the cartilage, sectioning the ear across the AP axis will not always capture these structures. Thus, we have further added serial sections of *short-ear* (Fig. S8) and het sibling P7 (Fig. S7) ears to further supplement our data from wildtype *Mus* and *Acomys* as these sections show vasculature and nerves in the foramina. Fig 2A-C show foramen at different developmental time points and based on the timing of chondroprogenitor specification (Fig. 2G-H), suggest that the cartilage and vasculature form in parallel.

The spiny mouse is very interesting due to its regenerative capacity, but very little extra information is found in the analysis. The comparison of the development of the pinna in both mouse species is nice but descriptive. For example, relative timing of hair follicle and pinna development are shown but its unclear that there is any link.

We agree that our findings in the spiny mouse do not vary much from those in *Mus*.
However, the finding that the process of tissue differentiation is conserved between lab mouse and spiny mouse despite their different gestation length and developmental stage at parturition is important and our study presents strong, cellular data to support this. Moreover, we have expanded our molecular analysis in spiny mice to demonstrate the expansion of Sox9+ chondroprogenitors and timing of proliferation relative to differentiation of elastic cartilage (Fig. 2I-J, M). If regeneration in the spiny mouse redeploys developmental processes, this study provides the foundation to examine these processes in temporal and spatial detail.

The paper then examines the phenotype of the short ear mouse which has a mutation in Bmp5. The mice are found to affect a subset of pinna cartilage with more adipocytes. Lineage tracing was not performed so the relationship of these cell types is unclear and the authors hypothesise a role for mechanical force in postnatal accumulation of adipocytes, but this is not tested. The link with defects and foramen location is interesting but rather tenuous. It’s very unclear from Figure 5 whether there is any relationship. Similarly, the presence of perichondrium but no cartilage requires more analysis to strengthen this claim. Fig6G appears to show a gap in tenascin-C.

We appreciate that lineage tracing would be required to truly demonstrate that presumptive cartilage has not been mis-specified into adipose. However, we present new data to further support that mis-specification is not occurring. 1) Absence of chondroprogenitors can be observed as early as P5, but no increase in adipoblasts is evident even as late as P17 (Fig. 5B’ and 6D’-E’).
2) The spatial distribution of developing adipose at P17 and in adults is very different from where chondroprogenitors would reside (laterally vs centrally) (Fig. 4E-G and 6D’-E’). 3) There is a significant decrease in chondroblast proliferation, indicating this deficit is responsible for the *short ear* pinna phenotype (Fig 6A-C).
The persistence of the perichondrium has been clarified in the text and Fig. 5E-F. It appears to develop along the PD axis of the ear and can be seen collapsing on itself at P7.
However, the absence of the perichondrium at P17 suggests it fails to persist without chondroblasts/cytes. The text has been modified to reflect these observations which are based on TNC staining. To our knowledge, it has not been previously established that the developing perichondrium expresses TNC.
We have removed our hypothesis regarding the role of mechanical force from the figures.
It is still briefly discussed in the discussion section.

© 2025. Published by The Company of Biologists under the terms of the Creative Commons Attribution License (https://creativecommons.org/licenses/by/4.0/).

Overall, this paper needs a major rewrite to focus on the new. The title and abstract need to be completely re written with a focus on the short ear mutant, and more analysis would be required.

As per the reviewer's request/comments/suggestions, we have made every effort to strengthen our manuscript as detailed above.

***** Reviewer 3 Advance Summary and Potential Significance to Field:
In this work, Allen and colleagues show that pinna development is largely conserved within murid species and employ lineage tracing experiments to define the origin of all cells within the ear pinna of mice at late gestational and postnatal stages. In addition, they investigate the effect of loss of BMP5 on pinna development and find that the elastic cartilage is likely not formed properly in the distal pinna due to a defect in chondroprogenitor proliferation. This defect persists into adulthood where the regenerative potential of the cartilage in the distal pinna is impaired.
This study employs a combination of different histological and immunohistochemistry analysis of ear pinna sections from Mus and Acomys at a range of late gestational and postnatal stages and compares late stages of chondrogenesis in Bmp5+/se versus Bmp5se/se short ear mutant animals. This study is well executed, the data are convincing, and provide novel insights into normal pinna development, especially at postnatal stages, as well as into the role of Bmp5 in pinna chondrogenesis. This study will be a very good reference for the specialists in the field.

However, there are several aspects that require additional information and revision before publication to ensure that the advances provided are fully supported by the presented experimental evidence.

Thank you.

Reviewer 3 Comments for the Author:
Major revision issues
1. It is unclear if the pinna is ever fused to the head in Acomys as it is already not fused to the head at E20. Sections showing the fusion at earlier stages would highlight the similarities between ear pinna development in Mus and Acomys.

Due to the long gestation and difficulty estimating time of fertilization, we were unable to obtain samples of a fully fused *Acomys* ear. Even at E15 the epidermis has started separating between the head and ear. Although it is folded over on itself at this point. We agree this data would be valuable to obtain. Based on the similarities between the two species in other parts of the developmental sequence, we there is no reason not to believe that *Acomys* ears are also fused to the hear early in development.

2. The authors describe that chondrogenesis starts at P3 in Mus (line 158). Chondrogenesis starts with the condensation of mesenchymal cells, which starts already between E12.5 and E14.5 in the pinna of Mus. What the authors observe at P3 may be the onset of differentiation of chondroprogenitor cells into chondrocytes. This should be amended in the text and schematics (Fig. 2O and 3L).

Thank you for pointing out this detail. We agree that our results address cartilage differentiation rather than chondrogenesis and have updated the text and figures to reflect this. Based on our data the condensing mesenchymal cells are specified to SOX9+ chondroprogenitors at P1 and subsequently mature into cartilage.

3. The authors describe that Tnc secretion starts at P3 in Mus (line 158), but do not show any data to support this claim. Additional markers for perichondrium and chondroprogenitor differentiation should be tested to prove that chondrocytes in the pinna mature at this stage, such as Col2b, Col9, and Aggrecan. Some of these may also show signal in Acomys pinna in contrast to Tnc.

Data showing TNC has been removed from our developmental time course. TNC stains are still in our *short ear* studies (Fig. 5E-F'). To observe chondroprogenitor specification we have used SOX9 but were unable to obtain successful staining using the suggested markers. Our analysis of

© 2025. Published by The Company of Biologists under the terms of the Creative Commons Attribution License (https://creativecommons.org/licenses/by/4.0/). 

wildtype perichondrium development is now just limited to histological observations (Fig. 2).

4. A reference linking TNC expression to perichondrium is missing.

To our knowledge, the expression of TNC in developing perichondrium has not been previously established. We have modified the text to clarify that TNC staining overlaps with presumptive perichondrium (lines 294-301) but have not shown its co-localization with other markers.

5. Line 186: "…due to precocial development in Acomys, pre-cartilaginous mesenchymal condensation occurred in utero as did the beginning phases of hair follicle morphogenesis (Fig. 3A-F)…" The authors should note that pre-cartilaginous mesenchymal condensation also occurs in utero in Mus (Minoux et al. 2013, Zhang et al. 2020,…)

This has been added (line 137)

6. Line 189: "…Of note, unfolding occurred after myogenesis but prior to apparent hair follicle morphogenesis and chondrogenesis, making this the only developmental benchmark that occurs in an order different from Mus…" Please discuss how this statement fits with the findings in Zhang et al. 2020 showing the presence of differentiated muscle cells in the pinna of Mus as early as E14.5.

This statement has been corrected as additional analysis has revealed in both species the order of differentiated tissue appearance is conserved. The text has been modified to clarify that our developmental time course establishes when differentiating tissues are histologically identifiable. In this case, we first identified striated skeletal muscle at P1 (*Mus*) and E20 (*Acomys*) (Fig. 2B and H).

7. Considering that the expression of BMP4 in the pinna at embryonic stages is located at the base and distal tip of the pinna (Minoux et al. 2013), the authors may discuss the possibility that BMP4 might partially rescue the effect of loss of BMP5.

We completely agree and have added this to the discussion (lines 438-439)

8. The hypothesis that the foramina correspond to borders of independently maturing areas of chondrogenesis is not strongly supported by the data shown. Does this statement hold true throughout the whole pinna? Do the locations of the sections along the pinna match in BMP5+/se and BMP5se/se animals for the images shown? Do the calculations in panel 6C and 6I take into account the fact that the pinna in BMP5se/se is shorter than in heterozygous animals?

We appreciate this point which led us to examine cartilage development and differentiation in much greater detail. First, our previous statement does not hold true through the whole pinna, as evidenced by our new serial sections (Fig. S7 and S8). The sectioned ears originally presented were obtained from the approximate middle of the ear, but likely are not in exactly the same place. It seems we by chance obtained several sections with similar foramen distribution. The calculations have been removed as we no longer believe there is a correlation between the missing cartilage and foramen locations.

9. EDU tracing experiments or further Ki67 staining at different late gestational and postnatal stages would further support the finding that elastic cartilage does not develop properly due to a proliferation defect of chondroprogenitors in BMP5se/se animals.

We have added additional KI67 stains at earlier time points to establish when proliferation in the chondroprogenitors begins. We do not believe later timepoints would be informative as the chondroprogenitors appear to be largely absent then, so the deficiency has already been established.

Minor points
1. Line 136: "…the ear pinna unfolds during embryonic development in Acomys (Fig. 1F)…" There is no Acomys shown in Fig. 1F. Maybe cite Fig. S2 and S3 here instead of showing different timing of pinna unfolding.

© 2025. Published by The Company of Biologists under the terms of the Creative Commons Attribution License (https://creativecommons.org/licenses/by/4.0/). 

Citation added (line 163)

2. Line 148: It is unclear where the stratum corneum, stratum spinosum, and stratum basale are located in Fig. 2A.

The stratum spinosum and stratum basale are poorly differentiated at this stage (lines 138-140)

3. Line 152: "… At P1, condensing mesenchymal cells continued to form a dense aggregation while the dermal compartment of the pinna expanded resulting in decreased cellular density (Fig. 2B). …"..Fig 2B does not show data of P1 pinna.

Figure has been modified so reference is correct (line 140-142)

4. Line 166: should be Fig. 2J Corrected
5. Line 206: "…Previous work inactivating Hoxa2 in mice demonstrated the importance of neural crest migration through the first pharyngeal arch for pinna formation…" It should read: ' second pharyngeal arch for pinna formation.

This sentence has been removed

6. Line 235: part of the sentence is missing.

Sentence has been corrected (line 224)

7. Line 410: typo "analgen" and "from side" Line modified (line 391)
8. Line 440: typo "compared to other body tissues" This line has been removed for brevity

9. Line 449: "…connective tissue of the ear, whereas the muscle is derived from a different germ layer (likely mesoderm)…" please cite and discuss Zhang et al. 2020, as they have already shown with lineage tracing experiments that muscle cells in the pinna of Mus are not of neural crest origin.

Zhang study now is cited in the results (line 210) and discussion (line 417)

10. Line 590: should be Fig. 4

Reference is now correct (line 594)

11. All figures should have colorblind-friendly coloring. Avoid usage of e.g. green and red in the same panel.

We apologize and have done our best to address this point.

12. Immunostaining images of Fig. 5A and D are very dark and thus it is hard to see what the authors want to show.

All images have been brightened

13. In Fig. 2, 3, S2, and S3 the orientation and location of the sections in particular for embryonic stages should be more clearly specified.

We have done our best to address this.

14. In Fig. 2: the legend does not mention what the cyan arrows mean; the legend is missing the annotation for acronyms in panel A,B,C, and F.

Cyan arrow has been specified. Acronyms have been removed. (Fig. 2)

15. Fig. 3E and 3F are not mentioned in the text.

© 2025. Published by The Company of Biologists under the terms of the Creative Commons Attribution License (https://creativecommons.org/licenses/by/4.0/).

3E is now 2J and is mentioned (Line 163-164). Fig 3 F has been removed.

16. Fig. 5: errors in the figure legend, e.g. "…Bmp5+/se (E) and Bmp5se/se (F)…" should be (I) and (J)

Corrected

17. In Fig. 7, what do white arrows point at?

White arrows indicate the limits of the cartilage gap. Added to figure legend.

18. Fig. 8: There are two panels labelled E. The figure legend for C and D need to be swapped. Images are very dark and it is hard to see the Sox9 signal.

Panel labels have been corrected. SOX9 signal has been brightened and a magnification of the injury sight has been added without the nuclear marker to aid in visualization of the SOX9 nodules.

19. Data shown in Fig. 8B contradicts the statement in the text (line 366).

Fig. 8B had a mistake that caused short-ear and het sibling data to get switched. Text is correct. Panel has been corrected (Fig 7B)

20. What is the green/yellow signal/channel in immunofluorescent images of Fig. 5, 6, 7, 8?

The 488nm channel was imaged to capture tissue autofluorescence. There is no specific marker. Epidermis and cartilage matrix can be resolved somewhat in this way, allowing better visualization of the histology. This also allows for identification of highly autoflourescent cells such as red blood cells and hair shafts which appear yellow due to detection in both the red and green channel.

21. In the author contribution section CKH is mentioned, but s/he is not listed as an author in the first page of the manuscript.

CHK has been removed from contributions.

---

Second decision letter

MS ID#: DEVELOP/2023/202474

MS Title: Neural crest derived chondroprogenitors require BMP5 to proliferate within the ear pinna

Authors: Robyn S Allen, Shishir Biswas, and Ashley W Seifert

**I have now received all the referees reports on the above manuscript, and have reached a decision. The referees' comments are appended below, or you can access them online: please go to BenchPress and click on the 'Manuscripts with Decisions' queue in the Author Area.**

**The overall evaluation is positive and we would like to publish a revised manuscript in Development, provided that the referees' comments can be satisfactorily addressed. Please attend to all of the reviewers' comments in your revised manuscript (see Editor's Note) and detail them in your point-by-point response. If you do not agree with any of their criticisms or suggestions explain clearly why this is so. If it would be helpful, you are welcome to contact us to discuss your revision in greater detail. Please send us a point-by-point response indicating your plans for addressing the referees' comments, and we will look over this and provide further guidance.**

**Editor's note:**

© 2025. Published by The Company of Biologists under the terms of the Creative Commons Attribution License (https://creativecommons.org/licenses/by/4.0/).

- **Please consider the suggestion of revising the Introduction and Discussion.**
- **Provide a discourse on the disparity of the findings of this study from the Yang et al study, in the context of cellular proliferative activity versus cellular shrinkage.**
- **Explain the apparent increase in Sox9 activity in the ear of the mutant relative to the control**

Reviewer 1

*Advance summary and potential significance to field*

The manuscript by Allen et al has two major threads. Firstly it describes a detailed ontogeny of murid ear development in an outbred Mus strain as well as in an Acomys species which has double the gestational period of Mus. Secondly, the manuscript describes the underlying developmental basis for the short ear phenotype in the se/se mouse, which is caused by homozygosity of a truncating allele of Bmp5. While this first section is necessarily descriptive, it nevertheless establishes the conserved nature of pinna development in murid species despite very distinct developmental timelines. While the Acomys data is not essential for the subsequent assessment of the developmental basis of the se/se mutant mouse line, it is still informative and novel data from a murid species which is frequently used for regenerative studies. The detailed analysis of pinna development in the se/se mutant has revealed some intriguing new aspects of pinna maturation that are noteworthy and of potential significance to the field, especially given how understudied pinna development is. Overall, this is a solid paper, that is clearly written and the results generally well presented. And the authors have responded well to the first round of critiques.

*Comments for the author*

The authors have responded very well to the first round critiques that consisted of numerous minor concerns as well as some more significant issues. In this regard all of my issues have been satisfactorily addressed. The only new comment I have relates to Fig 5D/5D' - the se/se mutant appears to have significantly increased Sox9 staining at P17 marking follicles compared to the control, yet this is not commented on. At the earlier stage, P7, there does not appear to be a difference but this is only two days after hair follicle development begins. Is this observation at P17 consistent and does this also suggest, like adipocyte number, hair follicle number expands, either as a direct or indirect effect of loss of Bmp5?

Reviewer 2

*Advance summary and potential significance to field*

A very useful comparative analysis of pinna development in two murine models combined with an analysis of the role of Bmp5 in the pinna.

*Comments for the author*

The paper has been extensively revised along the lines suggested by the reviewers. New data has been added and other confirmatory data moved to supplementary files. This has much improved the flow of the paper and helped to unite the different sections. The abstract is now much more comprehensive. Importantly, some previous findings were not backed up after further analysis and have been removed. This highlights the importance of the review process.
Since this paper was last reviewed a further paper has been published in eLife on the role of Bmps in auricular chondrocytes by another group, which has some similar findings and highlights the spatial role of BMPs in the pinna using different mouse models (Yang et al., 2024). However, this paper has some very
different conclusions, for example that microtia is caused by shrinkage of chondrocytes rather than reduced proliferation after loss of Bmps. The differences between the two papers are discussed to some degree (although not in the context of proliferation changes which are a major report of this paper) so there could be more comparison and explanation of the differences. The discussion

© 2025. Published by The Company of Biologists under the terms of the Creative Commons Attribution License (https://creativecommons.org/licenses/by/4.0/). 

around fate changes and adipogenesis is now much clearer. The Yang paper highlighted fate changes to osteoblasts, so the fact that this was not observed in the Bmp5 mutant should be discussed.

I still think the title could more reflect the whole paper as Figure 1-3 are an interesting comparative study, and 4-7 are focused on the Bmp5 mutant (with Figure 8 being a schematic to tie everything together). The title is focused on the findings of the Bmp mutant and misses the opportunity to mention the comparative aspect. A slightly broader title would also help to distinguish this paper from the Yang paper.

Reviewer 3

*Advance summary and potential significance to field*

In this work, Allen and colleagues show that pinna development is largely conserved within murid species and employ lineage tracing experiments to define the neural crest contribution to the cells within the ear pinna of mice at late gestational and postnatal stages. In addition, they investigate the effect of loss of BMP5 on pinna development and find that the elastic cartilage is likely not formed properly in the distal pinna due to a defect in chondroprogenitor proliferation. This defect persists into adulthood where the regenerative potential of the cartilage in the distal pinna is impaired.
This study employs a combination of different histological and immunohistochemistry analysis of ear pinna sections from Mus and Acomys at a range of late gestational and postnatal stages and compares late stages of chondrogenesis in Bmp5+/se versus Bmp5se/se short ear mutant animals. This study is well executed and provides novel insights into normal pinna development, especially at postnatal stages, as well as into the role of Bmp5 in pinna chondrogenesis. It will be an important reference for the specialists in the field.

While this revised version is much improved and the authors have addressed most of my previous concerns, two major issues remain for revision (see below) before the paper can be considered for publication.

*Comments for the author*

Reviewer 3 Advance Summary and Potential Significance to Field:
In this work, Allen and colleagues show that pinna development is largely conserved within murid species and employ lineage tracing experiments to define the neural crest contribution to the cells within the ear pinna of mice at late gestational and postnatal stages. In addition, they investigate the effect of loss of BMP5 on pinna development and find that the elastic cartilage is likely not formed properly in the distal pinna due to a defect in chondroprogenitor proliferation. This defect persists into adulthood where the regenerative potential of the cartilage in the distal pinna is impaired.

This study employs a combination of different histological and immunohistochemistry analysis of ear pinna sections from Mus and Acomys at a range of late gestational and postnatal stages and compares late stages of chondrogenesis in Bmp5+/se versus Bmp5se/se short ear mutant animals. This study is well executed and provides novel insights into normal pinna development, especially at postnatal stages, as well as into the role of Bmp5 in pinna chondrogenesis. It will be an important reference for the specialists in the field.

While this revised version is much improved and the authors have addressed most of my previous concerns, two major issues remain for revision (see below) before the paper can be considered for publication.

Reviewer 3 Comments for the Author:
Major revision issues:

1. In Introduction: ' Forward genetic studies have determined that the ear pinna is derived from Hoxa2+ neural crest cells, though the exact sources of these cells within the pharyngeal arches is

© 2025. Published by The Company of Biologists under the terms of the Creative Commons Attribution License (https://creativecommons.org/licenses/by/4.0/). 

debated (Cox et al., 2014; Green and Green, 1942; Mallo and Gridley, 1996; Minoux et al., 2013; Palmer et al., 2016; Rijli et al., 1993; Theiler and Sweet, 1986b).' This sentence is inaccurate, since in mouse the genetic fate mapping of the pinna clearly assessed its origin from Hoxa2+ neural crest derived mesenchyme of the second pharyngeal arch (Minoux et al., 2013). Moreover, loss- and gain-of function studies have shown that Hoxa2, expressed in second pharyngeal arch neural crest, is both necessary and sufficient to drive pinna morphogenesis (Rijli et al., 1993; Santagati et al., 2005; Minoux et al., 2013). THE ABOVE SENTENCE MUST BE THEREFORE REVISED AND MAY BE REPHRASED TO: 'In humans, the Hillocks of His contribute to the pinna components, though the exact sources of these cells within the pharyngeal arches is debated (Cox et al., 2014; Green and Green, 1942; Palmer et al., 2016; Theiler and Sweet, 1986b). In mouse, genetic fate mapping (Minoux et al., 2013) as well as loss and gain of function studies (Mallo and Gridley, 1996; Minoux et al., 2013; Rijli et al., 1993) demonstrated that the ear pinna, with the possible exception of the tragus, is derived from the Hoxa2+ cranial neural crest cells of the second pharyngeal arch and that, in these cells, Hoxa2 is required for pinna morphogenesis.'

We thank the reviewer for their corrections to the accuracy of the introduction. The suggested edits have been added (lines 58-65).

2. In Discussion: 'Previous work showed that Rhombomere4- and Hoxa2-expressing neural crest cells (NCC) of the second pharyngeal arch give rise to some or all of the embryonic pinna and that Hoxa2 controls pinna morphogenesis via regulation of Bmp4 and Bmp5 (Minoux et al., 2013).' Again, this sentence is inaccurate (see point 1). THE SENTENCE MUST BE REVISED AND MAY RATHER READ: ' Previous work showed that Rhombomere4-derived and Hoxa2-expressing cranial neural crest cells (NCCs) of the second pharyngeal arch give rise to the embryonic pinna, with the possible exception of the tragus, and that Hoxa2 controls pinna morphogenesis via regulation of Bmp4 and Bmp5 (Minoux et al., 2013).'

We thank the reviewer for their suggestions to improve the accuracy of our discussion. The suggested edits have been added. (lines 392-395)

Minor issues:
- Please check carefully typos throughout the manuscript
- In Discussion: ' We sought to ask whether additional tissues of the adult pinna arose from non-NCCs migration during later stages of pinna morphogenesis.' SHOULD RATHER READ: ' We sought to ask whether additional tissues of the adult pinna arose from non-NCC migration during later stages of pinna morphogenesis.' or ' We sought to ask whether additional tissues of the adult pinna arose from migration of non-NCCs during later stages of pinna morphogenesis.'
- in Discussion: ' Using lineage analysis of NCCs in mice we confirm that NC cells..' SHOULD RATHER READ: ' Using lineage analysis in mice we confirm that NCCs...'

---

**Second revision**

****

Editor’s note:

- Please consider the suggestion of revising the Introduction and Discussion.
- Provide a discourse on the disparity of the findings of this study from the Yang et al study, in the context of cellular proliferative activity versus cellular shrinkage.
- Explain the apparent increase in Sox9 activity in the ear of the mutant relative to the control

These points have all been addressed in the revised manuscript. See detailed response below.

An additional notable change has been made to Fig. S11, which now includes details of repeated measures ANOVA and post-hoc analysis on ear hole closure.

----------------------

© 2025. Published by The Company of Biologists under the terms of the Creative Commons Attribution License (https://creativecommons.org/licenses/by/4.0/).

Reviewer 1 Advance Summary and Potential Significance to Field: The manuscript by Allen et al has two major threads. Firstly, it describes a detailed ontogeny of murid ear development in an outbred Mus strain as well as in an Acomys species which has double the gestational period of Mus. Secondly, the manuscript describes the underlying developmental basis for the short ear phenotype in the se/se mouse, which is caused by homozygosity of a truncating allele of Bmp5. While this first section is necessarily descriptive, it nevertheless establishes the conserved nature of pinna development in murid species despite very distinct developmental timelines. While the Acomys data is not essential for the subsequent assessment of the developmental basis of the se/se mutant mouse line, it is still informative and novel data from a murid species which is frequently used for regenerative studies. The detailed analysis of pinna development in the se/se mutant has revealed some intriguing new aspects of pinna maturation that are noteworthy and of potential significance to the field, especially given how understudied pinna development is. Overall, this is a solid paper, that is clearly written and the results generally well presented. And the authors have responded well to the first round of critiques.

Reviewer 1 Comments for the Author:
The authors have responded very well to the first round critiques that consisted of numerous minor concerns as well as some more significant issues. In this regard all of my issues have been satisfactorily addressed. The only new comment I have relates to Fig 5D/5D' - the se/se mutant appears to have significantly increased Sox9 staining at P17 marking follicles compared to the control, yet this is not commented on. At the earlier stage, P7, there does not appear to be a difference but this is only two days after hair follicle development begins. Is this observation at P17 consistent and does this also suggest, like adipocyte number, hair follicle number expands, either as a direct or indirect effect of loss of Bmp5?

**RES:** We agree that short ear mutants appear to have brighter staining in the follicles/sebaceous glands. Upon further investigation we found that hair follicle density is similar between short ear mutants and their het siblings. Follicle density may appear higher in the short ear mutant due to more proximodistal compression of tissues (following failure to lengthen). Differences in staining intensity cannot be quantitatively assessed due to lack of appropriate fluorescent standards but the subjective appearance has been noted in the text. (See lines 269-271, 275-276, and Fig S7).

***** Reviewer 2 Advance Summary and Potential Significance to Field:
A very useful comparative analysis of pinna development in two murine models combined with an analysis of the role of Bmp5 in the pinna.

Reviewer 2 Comments for the Author:
The paper has been extensively revised along the lines suggested by the reviewers. New data has been added and other confirmatory data moved to supplementary files. This has much improved the flow of the paper and helped to unite the different sections. The abstract is now much more comprehensive. Importantly, some previous findings were not backed up after further analysis and have been removed. This highlights the importance of the review process. Since this paper was last reviewed a further paper has been published in eLife on the role of Bmps in auricular chondrocytes by another group, which has some similar findings and highlights the spatial role of BMPs in the pinna using different mouse models (Yang et al., 2024). However, this paper has some very different conclusions, for example that microtia is caused by shrinkage of chondrocytes rather than reduced proliferation after loss of Bmps. The differences between the two papers are discussed to some degree (although not in the context of proliferation changes which are a major report of this paper) so there could be more comparison and explanation of the differences. The discussion around fate changes and adipogenesis is now much clearer. The Yang paper highlighted fate changes to osteoblasts, so the fact that this was not observed in the Bmp5 mutant should be discussed.

**RES:** The observations by Yang et al. have been expounded upon in the discussion. That study carried out its experiments in young and adult mice, after the auricular cartilage had been established. With our data, these results show that BMP signaling is important for chondrocyte proliferation during ear growth (starting around P5), and important for chondrocyte maintenance after development (P21+). This pattern is in keeping with the multiple roles BMP plays in development and maintenance of other cartilaginous tissues. Additional differences between *short*

© 2025. Published by The Company of Biologists under the terms of the Creative Commons Attribution License (https://creativecommons.org/licenses/by/4.0/). 

*ear* mutants and pinna BMPR1 KO mice, likely result from knocking out a receptor, and as a consequence all BMP signaling, rather than one of many BMP ligands. (see lines 414-422)

I still think the title could more reflect the whole paper as Figure 1-3 are an interesting comparative study, and 4-7 are focused on the Bmp5 mutant (with Figure 8 being a schematic to tie everything together). The title is focused on the findings of the Bmp mutant and misses the opportunity to mention the comparative aspect. A slightly broader title would also help to distinguish this paper from the Yang paper.

**RES:** We appreciate the suggestion. The title has been modified per the reviewer's suggestion

***** Reviewer 3 Advance Summary and Potential Significance to Field:
In this work, Allen and colleagues show that pinna development is largely conserved within murid species and employ lineage tracing experiments to define the neural crest contribution to the cells within the ear pinna of mice at late gestational and postnatal stages. In addition, they investigate the effect of loss of BMP5 on pinna development and find that the elastic cartilage is likely not formed properly in the distal pinna due to a defect in chondroprogenitor proliferation. This defect persists into adulthood where the regenerative potential of the cartilage in the distal pinna is impaired.

This study employs a combination of different histological and immunohistochemistry analysis of ear pinna sections from Mus and Acomys at a range of late gestational and postnatal stages and compares late stages of chondrogenesis in Bmp5+/se versus Bmp5se/se short ear mutant animals. This study is well executed and provides novel insights into normal pinna development, especially at postnatal stages, as well as into the role of Bmp5 in pinna chondrogenesis. It will be an important reference for the specialists in the field.

While this revised version is much improved and the authors have addressed most of my previous concerns, two major issues remain for revision (see below) before the paper can be considered for publication.

Reviewer 3 Comments for the Author:
Major revision issues:

1. In Introduction: ' Forward genetic studies have determined that the ear pinna is derived from Hoxa2+ neural crest cells, though the exact sources of these cells within the pharyngeal arches is debated (Cox et al., 2014; Green and Green, 1942; Mallo and Gridley, 1996; Minoux et al., 2013; Palmer et al., 2016; Rijli et al., 1993; Theiler and Sweet, 1986b).' This sentence is inaccurate, since in mouse the genetic fate mapping of the pinna clearly assessed its origin from Hoxa2+ neural crest derived mesenchyme of the second pharyngeal arch (Minoux et al., 2013). Moreover, loss- and gain-of function studies have shown that Hoxa2, expressed in second pharyngeal arch neural crest, is both necessary and sufficient to drive pinna morphogenesis (Rijli et al., 1993; Santagati et al., 2005; Minoux et al., 2013). THE ABOVE SENTENCE MUST BE THEREFORE REVISED AND MAY BE REPHRASED TO: 'In humans, the Hillocks of His contribute to the pinna components, though the exact sources of these cells within the pharyngeal arches is debated (Cox et al., 2014; Green and Green, 1942; Palmer et al., 2016; Theiler and Sweet, 1986b). In mouse, genetic fate mapping (Minoux et al., 2013) as well as loss and gain of function studies (Mallo and Gridley, 1996; Minoux et al., 2013; Rijli et al., 1993) demonstrated that the ear pinna, with the possible exception of the tragus, is derived from the Hoxa2+ cranial neural crest cells of the second pharyngeal arch and that, in these cells, Hoxa2 is required for pinna morphogenesis.'

We thank the reviewer for their corrections to the accuracy of the introduction. The suggested edits have been added (lines 58-65).

2. In Discussion: 'Previous work showed that Rhombomere4- and Hoxa2-expressing neural crest cells (NCC) of the second pharyngeal arch give rise to some or all of the embryonic pinna and that Hoxa2 controls pinna morphogenesis via regulation of Bmp4 and Bmp5 (Minoux et al., 2013).' Again, this sentence is inaccurate (see point 1). THE SENTENCE MUST BE REVISED AND MAY RATHER READ: ' Previous work showed that Rhombomere4-derived and Hoxa2-expressing cranial neural crest cells

© 2025. Published by The Company of Biologists under the terms of the Creative Commons Attribution License (https://creativecommons.org/licenses/by/4.0/). 

(NCCs) of the second pharyngeal arch give rise to the embryonic pinna, with the possible exception of the tragus, and that Hoxa2 controls pinna morphogenesis via regulation of Bmp4 and Bmp5 (Minoux et al., 2013).'

We thank the reviewer for their suggestions to improve the accuracy of our discussion. The suggested edits have been added. (lines 392-395)

Minor issues:
- Please check carefully typos throughout the manuscript
- In Discussion: ' We sought to ask whether additional tissues of the adult pinna arose from non-NCCs migration during later stages of pinna morphogenesis.' SHOULD RATHER READ: ' We sought to ask whether additional tissues of the adult pinna arose from non-NCC migration during later stages of pinna morphogenesis.' or ' We sought to ask whether additional tissues of the adult pinna arose from migration of non-NCCs during later stages of pinna morphogenesis.'
- in Discussion: ' Using lineage analysis of NCCs in mice we confirm that NC cells..' SHOULD RATHER READ: ' Using lineage analysis in mice we confirm that NCCs...'

The manuscript has been carefully reviewed for additional typos and the reviewer's suggestions have been incorporated into the discussion. (second minor issue – line removed; third minor issue corrected - see line 297)

---

<u>Third decision letter</u>

MS ID#: dev.204560R1

MS Title: Ear pinna growth and differentiation is conserved in murids and requires BMP signaling for chondrocyte proliferation

Authors: Robyn S. Allen, Shishir K. Biswas and Ashley W. Seifert

I am happy to tell you that your manuscript has been accepted for publication in Development, pending our standard publication integrity checks.

Reviewer 1

As stated in prior review comments, this manuscript makes a significant and novel contribution to ear development - highlighting both important aspects of conservation between diverse murid species as well as the important role of Bmp signaling through the use of the short ear mutant.

The authors have addressed all my concerns

Reviewer 2

In this revised version, the authors have thoroughly addressed all my previous concerns. This interesting study may now be suitable for publication.

© 2025. Published by The Company of Biologists under the terms of the Creative Commons Attribution License (https://creativecommons.org/licenses/by/4.0/).
